# *Ginkgo biloba*: A Leaf of Hope in the Fight against Alzheimer’s Dementia: Clinical Trial Systematic Review

**DOI:** 10.3390/antiox13060651

**Published:** 2024-05-27

**Authors:** Guilherme Lopes de Oliveira Pagotto, Livia Maria Oliveira dos Santos, Najwa Osman, Caroline Barbalho Lamas, Lucas Fornari Laurindo, Karina Torres Pomini, Leila M. Guissoni, Enzo Pereira de Lima, Ricardo de Alvares Goulart, Virginia M. C. Strozze Catharin, Rosa Direito, Masaru Tanaka, Sandra Maria Barbalho

**Affiliations:** 1 Department of Biochemistry and Pharmacology, School of Medicine, University of Marília (UNIMAR), Avenida Higino Muzzi Filho, 1001, Marília 17525-902, SP, Brazil; guilhermefgv@outlook.com.br (G.L.d.O.P.); marylivia3@gmail.com (L.M.O.d.S.); najwaosman.kh@gmail.com (N.O.); lucasffffor@gmail.com (L.F.L.); karinatorrespomini@gmail.com (K.T.P.); guissoni.campos@gmail.com (L.M.G.); enzopereiralima@outlook.com (E.P.d.L.); ricardogoulartmed@hotmail.com (V.M.C.S.C.); 2 Department of Gerontology, Universidade Federal de São Carlos, UFSCar, São Carlos 13565-905, SP, Brazil; carolinelamas@estudante.ufscar.br; 3 Department of Biochemistry and Pharmacology, School of Medicine, Faculdade de Medicina de Marília (FAMEMA), Marília 17519-030, SP, Brazil; 4 Postgraduate Program in Structural and Functional Interactions in Rehabilitation, Marília 17525-902, SP, Brazil; virgmcscatharin@outlook.com; 5 Laboratory of Systems Integration Pharmacology, Clinical & Regulatory Science, Research Institute for Medicines (iMed.ULisboa), Faculdade de Farmácia, Universidade de Lisboa, Av. Prof. Gama Pinto, 1649-003 Lisboa, Portugal; rdireito@ff.ulisboa.pt; 6 Faculty of Pharmacy, Universidade de Lisboa, Av. Prof. Gama Pinto, 1649-003 Lisboa, Portugal; 7 Danube Neuroscience Research Laboratory, HUN-REN-SZTE Neuroscience Research Group, Hungarian Research Network, University of Szeged (HUN-REN-SZTE), Tisza Lajos krt. 113, H-6725 Szeged, Hungary; 8 Department of Biochemistry and Nutrition, School of Food and Technology of Marília (FATEC), Marília 17500-000, SP, Brazil

**Keywords:** Alzheimer’s disease, cognitive dysfunction, *Gingko biloba*, EGb 761, terpenoids, flavonoids

## Abstract

Alzheimer’s disease (AD) is a stealthy and progressive neurological disorder that is a leading cause of dementia in the global elderly population, imposing a significant burden on both the elderly and society. Currently, the condition is treated with medications that alleviate symptoms. Nonetheless, these drugs may not consistently produce the desired results and can cause serious side effects. Hence, there is a vigorous pursuit of alternative options to enhance the quality of life for patients. *Ginkgo biloba* (GB), an herb with historical use in traditional medicine, contains bioactive compounds such as terpenoids (*Ginkgolides* A, B, and C), polyphenols, organic acids, and flavonoids (quercetin, kaempferol, and isorhamnetin). These compounds are associated with anti-inflammatory, antioxidant, and neuroprotective properties, making them valuable for cognitive health. A systematic search across three databases using specific keywords—GB in AD and dementia—yielded 1702 documents, leading to the selection of 15 clinical trials for synthesis. In eleven studies, GB extract/EGb 761^®^ was shown to improve cognitive function, neuropsychiatric symptoms, and functional abilities in both dementia types. In four studies, however, there were no significant differences between the GB-treated and placebo groups. Significant improvements were observed in scores obtained from the Mini-Mental State Examination (MMSE), Short Cognitive Performance Test (SKT), and Neuropsychiatric Inventory (NPI). While the majority of synthesized clinical trials show that *Ginkgo* biloba has promising potential for the treatment of these conditions, more research is needed to determine optimal dosages, effective delivery methods, and appropriate pharmaceutical formulations. Furthermore, a thorough assessment of adverse effects, exploration of long-term use implications, and investigation into potential drug interactions are critical aspects that must be carefully evaluated in future studies.

## 1. Introduction

Alzheimer’s disease (AD) was first described in 1906 by the German psychiatrist Aloysius Alzheimer. This disease is an insidious and progressive neurodegenerative disorder that gradually deteriorates memory and cognitive functions [1]. Not only is it the leading cause of dementia in the elderly population worldwide, but it is also a major cause of disability. Typically, it displays the characteristic symptoms of being 75 years old, with around 4% of cases being early and severe, affecting language and visuospatial abilities associated with memory. Indeed, the manifestations and indications of AD extend beyond mere memory impairments, encompassing challenges in executing routine activities, disrupted sleep patterns, and even alterations in personality and mood [2,3,4,5,6,7].

Macroscopically, there is an irreversible loss of 15 to 35 percent of encephalic mass, with significant damage to the white matter, as well as diffuse cortical atrophy and bilateral, symmetrical, and subsequent cerebral gyri narrowing and sulci enlargement, primarily affecting the hippocampus. These abnormalities occur due to the accumulation of β-amyloid protein toxic plaques in the nervous tissue’s extracellular matrix, hyperphosphorylated tau protein in neurofibrillary tangles, and apolipoprotein E, which is the main risk factor for the development of AD. These changes impair the number of neurons, dendritic branching, and synapse zones [3,8,9,10].

**Figure 1:**
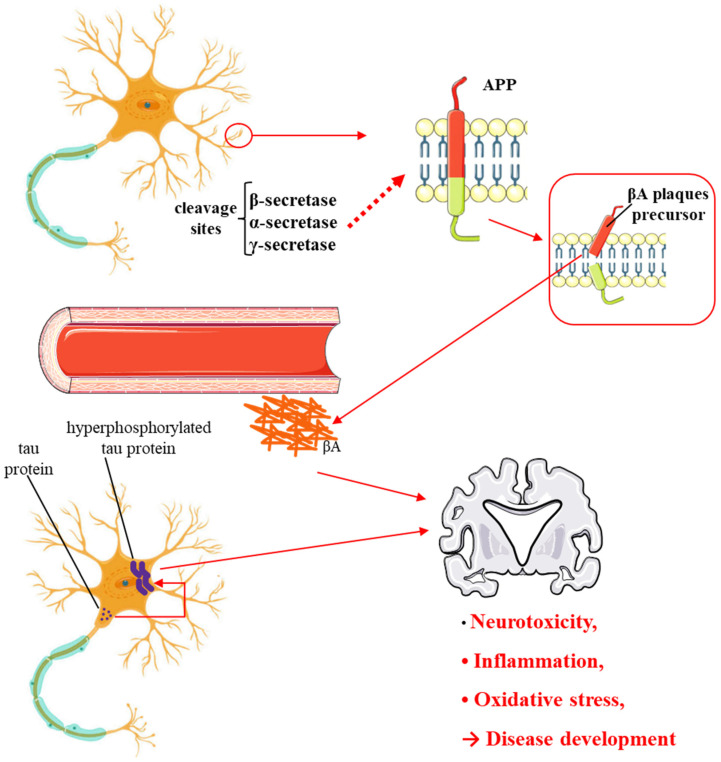
General mechanisms of damage in Alzheimer’s disease (AD). Many factors such as inflammation and free radicals can interfere with enzyme activation and the formation of βA plaques. This scenario leads to an increase in inflammatory factors’ release and the installation of oxidative stress, leading to neurotoxicity and AD development. APP—amyloid precursor protein; βA—β-amyloid.

The aggregation of the β-amyloid protein is thought to initiate all biochemical alterations in AD. It is a byproduct of the division (split) of amyloid precursor protein (APP), a membrane protein present in neurons. This reaction is catalyzed by three enzymes, alpha-secretase, beta-secretase, and gamma-secretase, each with a specific site of action on APP just above, slightly above, and within the phospholipid bilayer, respectively. The formation of B-amyloid protein toxic plaques is contingent upon the coordination of beta and gamma-secretases within the amyloidogenic pathway. Furthermore, tau protein, a microtubule-associated protein widely distributed inside axons that is responsible for the retrograde and anterograde transport of substances, becomes hyperphosphorylated and undergoes a shift associated with the generation of malformed helical filaments, which can then be found inside neurons’ soma and dendrites. These neurofibrillary tangles, as they are known, cause the nucleus of the parenchyma to be displaced, thereby preventing communication between parts of the cell and favoring their death and apoptosis [11,12,13,14,15,16] Figure 1 illustrates the general mechanisms related to Alzheimer’s disease.

Traditionally, AD has been treated with drugs that relieve symptoms, such as cholinesterase inhibitors (donepezil, rivastigmine, and galantamine) and N-methyl-D-aspartate receptor (NMDAR) antagonists (memantine). In AD, some neurons’ acetylcholine secretion is significantly reduced, and NMDAR is greatly stimulated, promoting a deleterious calcium influx sufficient to cause cell death and synaptic dysfunction. Thus, it is advantageous to prolong the action of the neurotransmitter’s remaining molecules attached to cholinergic receptors by preventing recycling and reducing excessive NMDAR activation [17]. Despite this, a cure for AD remains elusive because current pharmacotherapy cannot prevent APP enzymatic cleavage, the first step in the pathogenesis [18]. Hence, alternative treatments may improve patients’ quality of life with AD. Novel therapeutic opportunities for AD strategies include improving cognitive function and reducing amyloid deposition, opening up new treatment options, and including monoclonal antibodies [6,19,20,21,22,23,24,25].

The therapeutic strategies of herbal medicine are one of the available options for alleviating symptoms [26]. *Gingko biloba* (GB) is one of the plants whose extracts can be used as an adjuvant in the treatment of AD [27,28]. GB is a gymnosperm from Japan, China, and Korea that belongs to the *Ginkgo*aceae family and the *Ginkgo*opsida class of plants. For centuries, it has been used in popular medicine to treat a variety of health problems due to the abundance of bioactive substances it contains such as terpenoids (*Ginkgo*lides A, B, and C), polyphenols, organic acids, and flavonoids (quercetin, kaempferol, and isorhamnetin), which are associated with anti-inflammatory, antioxidant, and antiapoptotic effects [28,29]. Indeed, the standardized extract of GB (EGB 761) is a popular dietary supplement among the elderly to improve memory and prevent cognitive decline. These effects can be applied in the treatment of Alzheimer’s.

In the *Ginkgo* Evaluation of Memory Study (GEMS) (a longitudinal analysis), blood amyloid levels and dementia risk were assessed. This study examined the levels of beta-amyloid protein and other substances, such as vitamin B12, liver enzymes, and creatinine, in patients who had their Apolipoprotein E genotyped. For 8.5 years, the authors studied baseline β-amyloid (Aβ) levels in plasma and incident dementia in 2840 individuals aged 75 and up. From this total, 2381 were classified as cognitively normal, while 450 had mild cognitive impairment. The study found that higher plasma levels of Aβ1-40 and Aβ1-42 were associated with age, gender (women), low education, stroke history, hypertension, and creatinine levels. Normal subjects with dementia had lower levels of Aβ1-42 and Aβ1-42/Aβ1-40 compared to those without dementia. Aβ levels did not predict dementia in individuals with mild cognitive impairment, which is noteworthy [30].

This study aims to address the current issues in treating AD and dementia, where the efficacy of GB remains uncertain. Some studies found significant improvements in cognitive function, neuropsychiatric symptoms, and functional abilities, whereas others found no significant difference between the GB-treated and placebo groups. Given these inconsistencies, the study’s goal is to provide a comprehensive synthesis of existing clinical data, identify gaps in current research, and guide future studies. Furthermore, we set the objective to explore the potential of GB as an alternative treatment for AD and dementia, particularly considering the limitations and side effects of current AD medications. Through a systematic review and synthesis of clinical trials on the effects of GB extract/EGb 761^®^ on dementia patients, this study examines the effectiveness of GB’s bioactive compounds.

## 2. Methods

This systematic review aimed to investigate the potential benefits of GB for AD or other types of dementia. Only studies published in English were considered, sourced from MEDLINE–PubMed, EMBASE, and Cochrane databases. The search utilized MeSH terms including “*Ginkgo biloba*” and “Alzheimer’s disease” or “dementia,” or cognition. These terms guided the identification of studies examining GB’s effects on AD or dementia. Furthermore, we applied filters to enhance the quality of our search, namely “Clinical Trial” and “Randomized Controlled Trial”. Consequently, conferences, abstracts, letters to editors, and other emerging sources were evaluated but not included. Study identification and inclusion were carried out by S.M.B. and M.T., with conflicts resolved by a third author, M.T. Inclusion criteria were limited to human interventional studies, while exclusion criteria comprised studies not in English, reviews, editorials, case reports, poster presentations. and studies with animal models. The search for clinical trials had no temporal restriction. Data extraction followed the PICO (Population, Intervention, Comparison, and Outcomes) format, and study selection adhered to PRISMA (Preferred Reporting Items for Systematic Reviews and Meta-Analyses) guidelines [31,32]. The risk of bias in selected trials was evaluated using the Cochrane Handbook for systematic reviews of interventions [33].

## 3. Overview of the Included Studies and Resulting Findings

**Figure 2:**
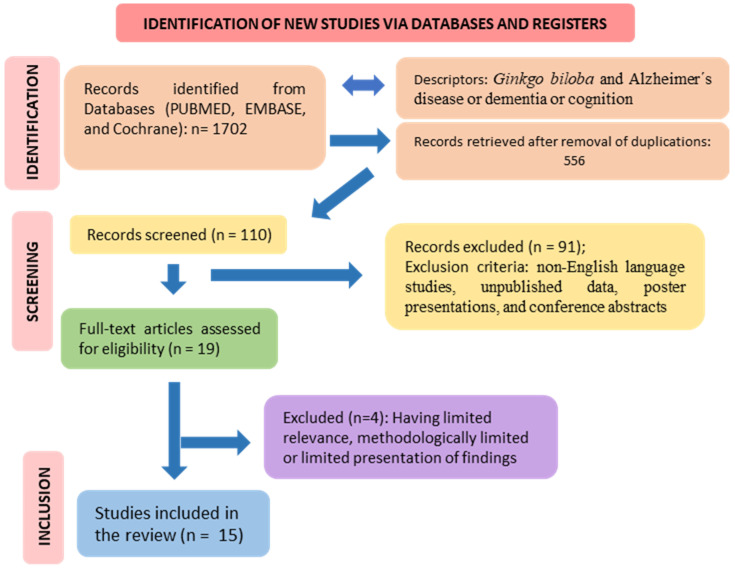
Flow diagram showing the study selection (PRISMA guidelines) [31,32].

Initially, 1702 studies were identified according to the search terms. After applying the inclusion/exclusion criteria, we identified 15 clinical trials involving the use of GB in AD and dementia that met these criteria. Figure 2 shows the selection of the studies in accordance with PRISMA guidelines. Fifteen studies compared the outcomes of GB or GB extract treatment to a placebo. The dosage ranged from 120 to 240 mg, with treatment periods ranging from four to 24 weeks. Four studies found no significant differences between groups treated with GB and placebo. Eleven studies found that administering GB extract/EGb 761^®^ improved cognitive function, neuropsychiatric symptoms, and functional abilities in both types of dementia. Significant differences were found in the Mini-Mental State Examination (MMSE), Short Cognitive Performance Test (SKT), and Neuropsychiatric Inventory (NPI) scores.

Table 1 lists the articles synthesized in this systematic review, while Table 2 displays the risk of bias assessments for the studies included. This systematic review reveals that GB can alleviate symptoms of Alzheimer’s.

**Table 1:** Effects of *Ginkgo biloba* in Alzheimer’s disease and dementia.

Reference	Model/Country	Population	Intervention/Comparison	Outcomes	Adverse Events
[34]	Randomized, double-blind, placebo-controlled study design in an outpatient setting/Egypt.	60 individuals complaining of memory impairment or forgetfulness and satisfying the clinical criteria for mild cognitive impairment, 27 ♂, 33 ♀, 50–80 years	The subjects were divided into 2 groups: G1 received one Memo capsule (combination of 750 mg of lyophilized royal jelly with standardized extracts of *GB* 120 mg) 1 timesd/4 weeks, and G2 received placebo.	Only the group treated with MEMO exhibited a statistically significant improvement in MMSE score after 4 weeks. The mean change in MMSE was +2.067 versus +0.133, respectively.	Neither group reported any serious AE (mild nausea, transient headache, and palpitation).
[35]	Randomized, parallel-group, double-blind, placebo-controlled GuidAge clinical trial/France.	2820 patients who spontaneously reported memory complaints, 940 ♂, 1880 ♀, 70 years or older.	Patients were divided into 2 groups: G1 received 120 mg standardized GB extract (EGb761) 2x/d, and G2 received placebo.	The study did not provide evidence for the protective effect of GB extract on the incidence of AD.	Both groups presented serious events such as death, stroke, bleeding, or cardiac disorders.
[36]	Randomized, double-blind clinical trial/Iran.	56 patients with primary degenerative dementia of the AD type, 51 completed the study, 23 ♂, 28 ♀, 50–75 years.	Patients were divided into G1 received GB (120 mg) 1x/d/24w, and G2 received rivastigmine (4.5 mg) 1x/d/24w.	This trial establishes GB efficacy and tolerability in Alzheimer’s dementia; however, it is not to the same level as rivastigmine.	Group 2: one adverse event was observed but not specified.
[37]	Randomized, controlled, double-blind, multi-center trial/Ukraine.	333 patients diagnosed with mild to moderate dementia AD, 113 ♂, 220 ♀, 50 years or more; and 71 patients diagnosed with VaD, 19 ♂, 52 ♀, 50 y or more.	Patients were divided into G1, received 240 mg of GB extract EGb 761^®^, 1x/d/24w and G2, which received placebo. Patients diagnosed with VaD were divided into two groups: G1 received 240 mg of GB extract EGb 761, 1x/d/24w, and G2 received placebo.	EGb 761^®^ improved cognitive function, neuropsychiatric symptoms, and functional abilities in both types of dementia. Significant differences were observed in SKT and NPI scores, and in most secondary outcomes, with no notable variations between AD and VaD subgroups.	Both groups presented headache, respiratory tract infection, increased blood pressure, and dizziness.
[38]	Multi-center, double-blind, randomized, placebo-controlled/Republic of Belarus, Republic of Moldova, and the Russian Federation.	410 patients suffering from mild to moderate AD or VaD; of those, 402 were counted into consideration, 123 ♂, 279 ♀, 50 years or more.	Patients were randomized into 2 groups: G1 received GB (240 mg) 1x/d/24w, and G2 received placebo.	After the interaction, G1 showed that EGb 761^®^ 1x/d at 240 mg effectively treats dementia, significantly improving cognitive performance and neuropsychiatric symptoms in patients (*p* < 0.001).	G1 and G2: headache, dizziness, respiratory tract infection, hypertension, somnolence; upper abdominal pain.
[39]	Multi-center, double-blind, randomized, parallel-group clinical trial/Ukraine.	410 individuals with probable AD (NINCDS-ADRDA criteria); possible AD with CVD (NINDS-AIREN criteria); or probable VaD (NINDS-AIREN criteria); (symptoms of dementia had to be present for at least 6 months, 132 ♂, 272 ♀, 50 years or older.	The population was divided into 2 groups; G1 received a once-daily tablet with 240 mg of EGb 761^®^; G2 received placebo. A screening period up to 4 weeks was necessary and was followed by a 24-week treatment period.	EGb 761^®^ was better than placebo for improving SKT and NPI total score (placebo group worsened in SKT and did not show alteration on the NPI total score).	G1 and G2 showed similar side effect rates (headache, respiratory tract infection, hypertension, dizziness).
[40]	Open, uncontrolled, clinical trial/Switzerland.	59 patients (DemTect score > 12, no obvious symptoms of dementia, and with the presence of at least two of the following symptoms: forgetfulness, impaired concentration, or impaired memory), 15 ♂, 44 ♀, 60 years or older.	All patients received 90 mg of fresh plant GB extract 2x/d/6w.	At the final visit, SF-12 mental score significantly increased from 48.3 ± 10.1 to 51.3 ± 7.9, but SF-12 body score (44.5 ± 9.2 to 45.3 ± 8.1) and DemTect score (15.9 ± 2.0 to 16.0 ± 2.3) did not change significantly. About half of the patients experienced memory and concentration improvement and fewer forgetfulness symptoms.	Twenty-seven patients reported 39 AE, which were not specified.
[41]	Randomized, double-blind exploratory trial/Bulgaria.	96 patients meeting the NINCDS-ADRDA criteria for probable AD, scored below 36 on the TE4D, below 6 on the CDT, between 9 and 23 on the SKT, and at least 5 on the 12-item NPI, 29 ♂, 65 ♀, 50 years or older.	Patients were divided in 3 groups: G1 received EGb 761 120 mg 2x/d; G2 received donepezil at a daily dose of 5 mg during the first 4 weeks and 10 mg for the remaining 18 weeks; G3 received both drugs at recommended doses. It was established a 22-week-treatment period.	During treatment, patients of 3 groups showed improvements over baseline values in all tests and rating scales. No statistically significant or clinically relevant differences could be detected between treatments.	26 AE were documented for 10 patients treated with EGb 761, 51 for 24 patients taking donepezil, and 29 for 18 patients receiving combined treatment. The most frequent were headache, insomnia, diarrhea, and fatigue.
[42]	Randomized, double-blind, placebo-controlled clinical trial/the US.	3069 with normal cognition (n = 2587) or MCI (n = 482) (impaired at or below the 10th percentile of Cardiovascular Health Study normative data, stratified by age and education, on at least 2 of 10 selected neuropsychological test scores from each cognitive domain, + CDR global score of 0.5), 1651 ♂, 1418 ♀, 75 years or older.	Patients were randomized into 2 groups: G1 received GB extract (120 mg) 2x/d, and G2 received placebo. There was a median follow-up of 6.1 years).	523 individuals with dementia (246 placebo, 277 GB); 92% classified as possible/probable AD or AD with brain vascular disease evidence. The rate of total dementia was 3.3 per 100 person-years in the GB group and 2.9 in placebo. GB had no effect on dementia or AD incidence in older people with normal cognition or MCI.	The AE profiles for the groups were similar, and there were no statistically significant differences in the rate of serious AE (death, coronary heart disease, stroke, bleeding).
[43]	A randomized, placebo-controlled, double-blind clinical trial/Ukraine.	400 patients with probable AD, possible AD with CVD or probable VaD, all of them with mild to moderate dementia as evidenced by a total score from 9 to 23 (both inclusive) on the SKT test battery, 110 ♂, 285 ♀, 50 years or older.	Patients were allocated either two tablets of EGb 761^®^ 120 mg or placebo per day for 22 weeks, being preceded by a medication-free screening period of up to 4 weeks.	The patients treated with EGb 761^®^ improved cognitive test performance regarding neuropsychiatric symptoms and activities of daily living; placebo deteriorated slightly on most of the outcome measures or remained unchanged, at best.	166 patients randomized to EGb 761^®^ reported 302 AE, and 178 patients treated with placebo reported 81 AE (headache, angina pectoris, dizziness, back pain).
[44]	Randomized, placebo-controlled, double-blind study/Italy.	76 patients with dementia of the Alzheimer type, 35 ♂, 41 ♀, aged 50–80 y.	Patients were randomized into 3 groups: G1 received GB 160 mg daily dose, G2 received donepezil 5 mg daily dose, and G3 was given placebo for 24 weeks.	Compared with the donepezil group, attention, memory, and cognitive performance (SKT test) showed a comparable important improvement.	The frequency of AE was very low (upper respiratory tract infection, dizziness, tinnitus, nausea).
[45]	Randomized, placebo-controlled, double-blind, parallel-group, multicenter trial/the US.	513 outpatients with uncomplicated dementia of the AD type scoring 10 to 24 on the MMSE and less than 4 on the modified HIS, without other serious health problems and not using continuous treatment to any psychoactive drug, 243 ♂, 270 ♀, 60 years or older.	Patients were divided into 3 groups: G1 received placebo, G2 received 120 mg of EGb 761^®^ per day (or twice-daily dose of 60 mg), and G3 received 240 mg of EGb 761^®^ per day (or twice-daily dose of 120 mg). They were accompanied for 26 weeks.	No differences between the treatment groups regarding cognitive endpoint. Placebo group did not worsen notably from baseline. At the same time, a similar slight decline was found for both actively treated groups.	The frequency of AE and serious AE was very low (upper respiratory tract infection, dizziness, tinnitus, nausea).
[46]	Randomized, double-blind, placebo-controlled, parallel-group, multicenter trial/the Netherlands.	214 participants with the diagnosis of dementia (AD or VaD) or age-associated memory impairment (AAMI). 19 ♂, 104 ♀, 50 y or older.	The subjects were randomly allocated to one of 3 treatments: G1: EGb 761^®^ 240 mg/d, G2: EGb 761^®^ 160 mg/d, and G3 placebo. After the 12-week treatment, the subjects were randomly separated in a second 12-week treatment period to continue their GB treatment or placebo.	No benefit of GB was observed. Small differences regarding SKT and CGI were found in GB group (no significant or clinically meaningful).	35 AE were registered (nausea, constipation, diarrhea, hospital entrance, and death)
[47]	Multi-center, double-blind, randomized, parallel-group, placebo-controlled trial/the US.	309 patients with a diagnosis of uncomplicated AD or multi-infarct dementia (ICD-10 and DSM-III-R criteria), 143 ♂, 166 ♀, 45 years or older.	Patients were randomly allocated to either G1 (that received EGb 40 mg 3x/d) or G2 (placebo) for 52 weeks following a 2-week, single-blind, placebo run-in period.	In comparison to the base-line values, placebo showed significant worsening in all domains of assessment; EGb slightly improved cognitive assessment and the daily living and social behavior.	Nausea, constipation, and diarrhea in both groups
[48]	Randomized, double-blind, placebo-controlled, parallel-group, multicenter trial/the Netherlands.	214 patients with a mild or moderate stage of dementia (as assessed by the results of SIDAM interview and by a score of 8–23 on the SKT), 34 ♂, 180 ♀, 50 years or older.	Patients were divided into 3 groups after a 3-week run-in period on placebo: G1 received EGb 761 120 mg 2x/d; G2 received twice-daily doses of EGb 761 80 mg; G3 continued receiving placebo. Outcomes were evaluated after 12 and 24 weeks of intervention.	In general, any remarkable shift in score on most of the outcome parameters during the intervention, either in the GB group or in the placebo	Dizziness, nervousness, and headache were the most common symptoms.

Abbreviations: AD: Alzheimer’s disease; ADRDA: Alzheimer’s Disease and Related Disorders Association; AE: Adverse events; AIREN: Association Internationale pour la Recherche et l’Enseignement en Neurosciences; CDT: Clock-Drawing Test; CDR: Clinical Dementia Rating; CGI: Clinical Global Impression; CVD: Cardiovascular Disease; DemTect: a brief screening test for dementia comprising five short subtests; DSM-III-R: Diagnostic and Statistical Manual of Mental Disorders; GB: *Gingko biloba*; HIS: Hachinski Ischemic Score; ICD-10: International Classification of Diseases; MCI: Mild Cognitive Impairment; MEMO: combination of 750 mg of lyophilized royal jelly with standardized extracts of GB 120 mg; MMSE: Mini-Mental State Examination; NAA: Nuremberg Gerontopsychological Rating Scale for Activities of Daily Living; NBACE: Neuropsychological Battery of Fundació ACE; NINCDS: National Institute of Neurological and Communicative Disorders and Stroke; NINDS: National Institute of Neurological Disorders and Stroke; NPI: Neuropsychiatric Inventory; SF-12: 12-item Short-Form Survey; SIDAM: Structured Interview For The Diagnosis of Dementia of the Alzheimer Type; SKT: Short Cognitive Performance Test; TE4D: Test For The Early Detection of Dementia With Discrimination from Depression; VaD: Vascular Dementia.

**Table 2:** Risk of bias assessment included in this review.

Study	Question Focus	Appropriate Randomization	Allocation Blinding	Double-Blind	Losses (<20%)	Prognostics or Demographic Characteristics	Outcomes	Intention to Treat Analysis	Sample Calculation	Adequate Follow-Up
[34]	Yes	Yes	Yes	Yes	Yes	Yes	Yes	NR	Yes	Yes
[35]	Yes	Yes	Yes	Yes	Yes	Yes	Yes	No	Yes	Yes
[36]	Yes	NR	Yes	Yes	Yes	Yes	Yes	No	NR	Yes
[37]	Yes	Yes	Yes	Yes	Yes	Yes	Yes	Yes	Yes	Yes
[38]	Yes	Yes	Yes	Yes	Yes	Yes	Yes	Yes	Yes	Yes
[39]	Yes	Yes	Yes	Yes	Yes	Yes	Yes	Yes	Yes	Yes
[40]	Yes	No	No	No	Yes	Yes	Yes	NR	NR	Yes
[41]	Yes	Yes	Yes	Yes	Yes	Yes	Yes	NR	NR	Yes
[42]	Yes	Yes	Yes	Yes	Yes	Yes	Yes	Yes	Yes	Yes
[43]	Yes	Yes	Yes	Yes	Yes	Yes	Yes	Yes	Yes	Yes
[44]	Yes	Yes	Yes	Yes	Yes	Yes	Yes	NR	NR	Yes
[45]	Yes	Yes	Yes	Yes	No	Yes	Yes	Yes	Yes	Yes
[46]	Yes	Yes	Yes	Yes	Yes	Yes	Yes	Yes	Yes	Yes
[47]	Yes	NR	NR	Yes	No	Yes	Yes	Yes	NR	Yes
[48]	Yes	Yes	Yes	Yes	Yes	Yes	Yes	Yes	Yes	Yes

Abbreviations: NR: not reported.

## 4. Ginkgo Biloba and Health Effects

### 4.1. Ginkgo Biloba, General Aspects

*Ginkgo*, also known as *GB*, is a tree that is notable for its distinctive characteristics. The earliest documented records of the *Ginkgo* tree date back to the 11th century in the Yangtze River region of China. It is now grown in a wide range of countries, including New Zealand, Brazil, the Netherlands, Chile, Japan, Belgium, Austria, England, Spain, Italy, Germany, France, Korea, Australia, and India, owing primarily to its ability to thrive in deep and sandy soils found in temperate and subtropical climate regions worldwide. In fact, *Ginkgo* is known for having a large genome and remarkable tolerance to various stress factors, whether abiotic or biotic; this species demonstrates remarkable resilience and adaptability in the face of adverse conditions, which contributes to its survival and longevity; additionally, it has the ability to cope with various forms of stress, whether caused by the physical environment or interactions with other organisms [49,50,51].

Its leaves, with their distinctive fan-shaped shape and green–yellow color, emit a strong odor. One notable feature of this tree is its impressive height, which ranges between 20 and 30 m. *Ginkgo* is also well known for its incredible longevity, which can last over 1000 years. The leaves are divided into two distinct lobes, hence the botanical name “*biloba*”. This unique feature makes the tree easily identifiable and distinct in its natural environment. Another interesting aspect is the *Ginkgo’*s reproductive system. This species’ trees have distinct male and female flowers, and instead of true seeds, the plant uses an outdated reproductive system that disperses “ovules” [49,52]. 

The first mention of the use of GB leaves for medicinal purposes appears in Wen-Tai’s text “Ben Cao Pin Hue Jing Yaor”, written in 1505 AD. This work provides information on the traditional uses of GB leaves. Over time, modern Chinese pharmacopeias have recognized the therapeutic properties of these leaves, particularly in the treatment of heart and lung disorders [53,54]. 

**Figure 3:**
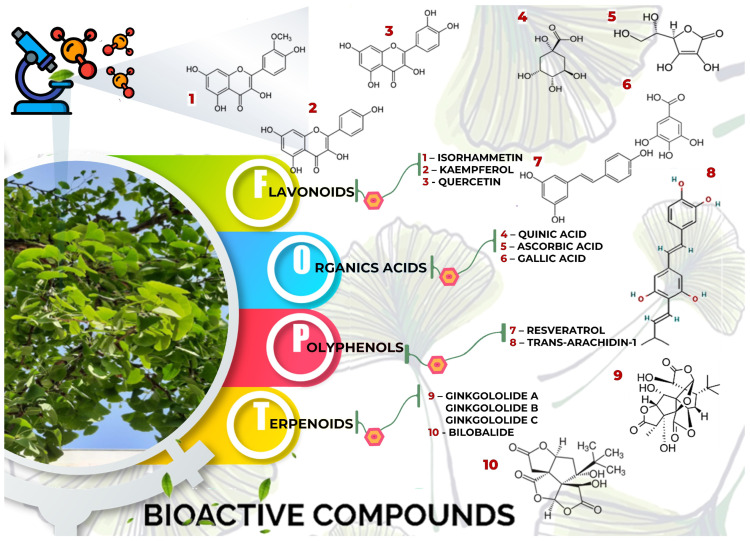
*Gingko biloba* tree, its bioactive compounds, classification, and chemical structures. *Gingko biloba* is rich in flavonoids, organic acids, polyphenols, and terpenoids. These compounds are responsible for the several biological effects of this plant.

*GB* contains several flavonoids, including quercetin, kaempferol, isorhamnetin, rutin, ginkgetina, and bilobetina. Terpenoids (*Ginkgo*lides) include *Ginkgo*lide A, *Ginkgo*lide B, *Ginkgo*lide C, *Ginkgo*lide J and *Ginkgo*lide M. Terpenoids (bilobalides) include bilobalide A and bilobalide B, as well as proanthocyanidins and organic acids like caffeic acid, *Ginkgo*lic acid, and quinic acid. The composition contains phytosterols, including β-sitosterol and stigmasterol. Figure 3 and Table 3 summarize the bio compounds composition of GB. The use of *Ginkgo* to improve symptoms in patients with oxidative inflammation-related diseases is thought to be associated with improved tissue perfusion and hypoxia tolerance. *Ginkgo*’s flavonoid glycosides exhibit antioxidant and anti-inflammatory properties, which can help reduce endothelial cell damage caused by free radical oxidation [28,55,56,57,58]. Figure 3 shows the major bioactive components found in this plant, parts of the *GB* tree, and the chemical compounds classified by class, and Table 1 lists the chemical structure of bioactive compounds, parts of the GB tree, and their effects. 

**Figure 4:**
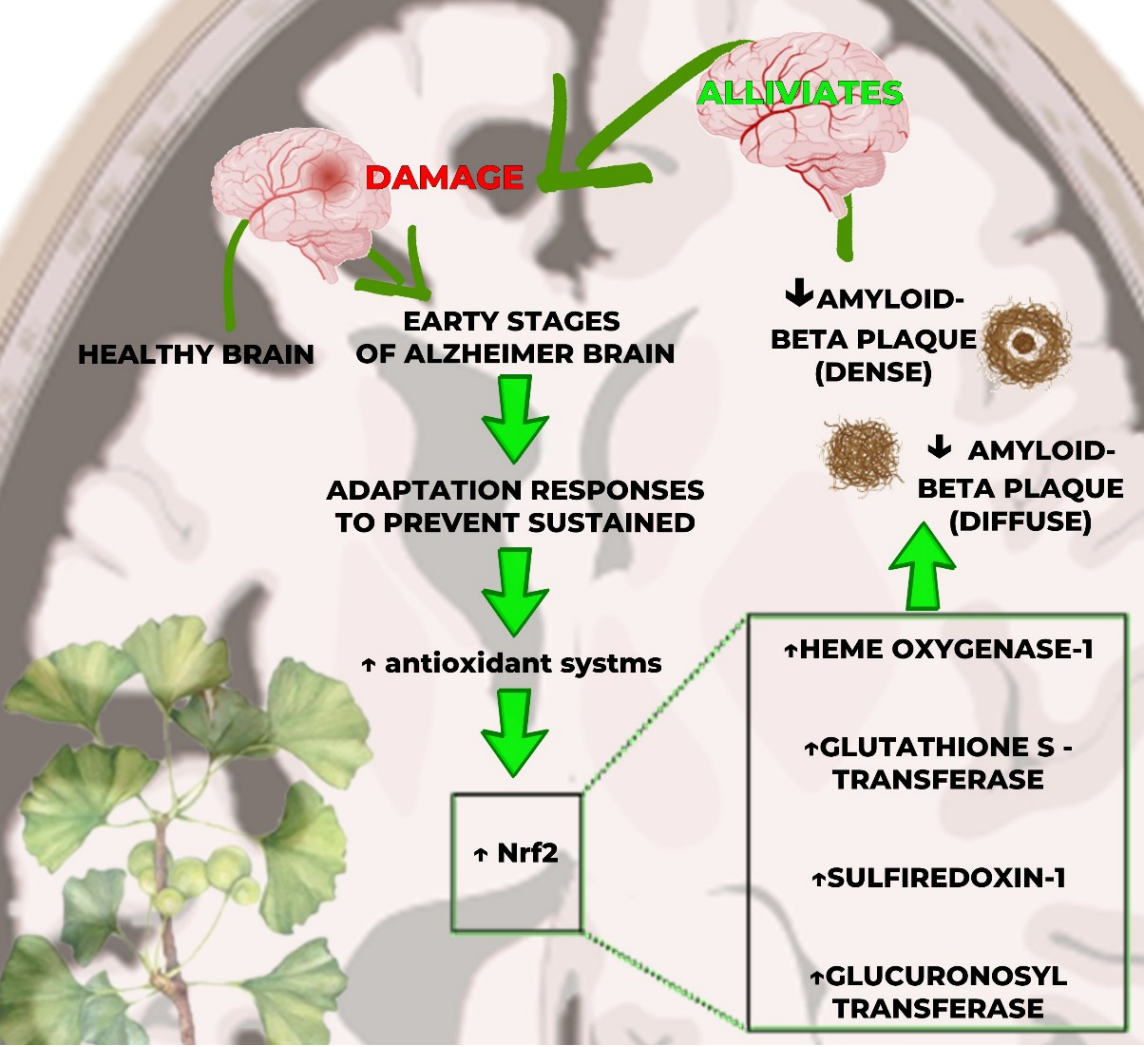
Hormesis and *Ginkgo biloba* activation of Nrf2 pathway for neuroprotection in Alzheimer’s disease (AD). Early stages of AD display adaptive responses involving light increase in Nrf2 gene expression, a transcriptional factor whose activation induces the transcription of genes encoding cytoprotective, antioxidant proteins that decrease brain damage by beta-amyloid deposits. *Ginkgo biloba*’s sulforaphane can upregulate Nrf2 by a hormetic dose response, thus reducing βA-induced cognitive decline and progression of the disease. ↑—increase; Nrf2—nuclear factor erythroid 2-related factor; SFN: sulforaphane; HO-1—heme oxygenate-1; GST—glutathione S-transferase; SRXN1—sulfiredoxin 1; UGT—UDP-glucuronosyltransferase; βA—beta-amyloid. ↓: decrease; ↑: increase.

**Figure 5:**
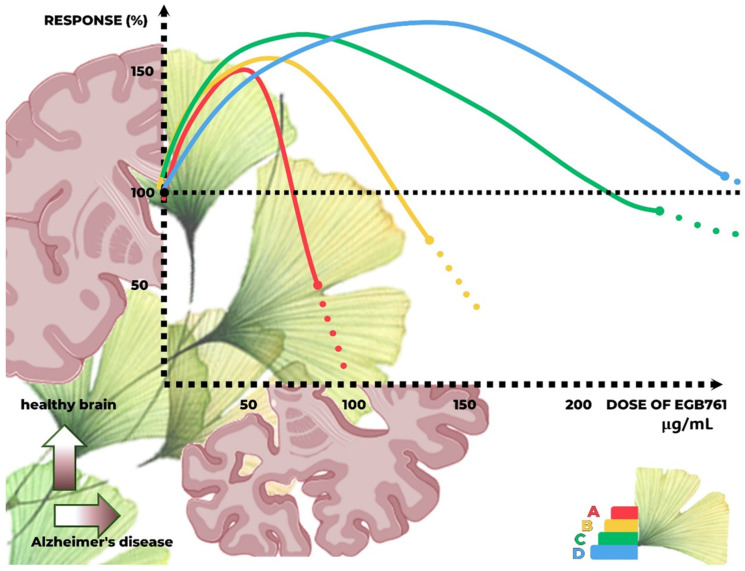
Some hormetic dose responses to EGb 761, according to previous studies. A. Effect of Ginkgo on viability and proliferation of mouse cochlea neural stem cells in vitro. B. Effect of Ginkgo on Sprague Dawley rat bone marrow mesenchymal stem cells survival to apoptosis in vitro. C. Effect of pretreatment Ginkgo on cell viability of H2O2-treated SH-SY5Y cells. D. Effect of Ginkgo on proliferation of MCF-7 AROM breast cancer cells. Hormetic ranges and toxic doses are different, depending on the type of model used in the study and measured endpoints. The graphic exhibits approximate doses of EGb 761 in µg/mL and their equivalent responses when compared to the control group. μg/mL: microgram/milliliters.

Furthermore, GB extracts are shown to induce biphasic dose responses in different cell types and endpoints. The magnitude of the dose–response is similar to the hormetic dose response that occurs through the stimulatory process, showing resistance within an adaptive temporal framework and repeated evaluated protocols. GB dose–responses seem to reflect the general occurrence of hormetic pathways that are independent of the endpoint, biological model, or mechanism. These findings have crucial implications for the study designs, dose selection and spacing, and sample size, illustrating the need to characterize the low-dose stimulatory response, as well as the optimal dose. Regarding polyphenols, they can promote antioxidant or pro-oxidant cytotoxic actions depending on concentration. At high doses, polyphenols can inhibit Nrf2 pathways and the expression of several antioxidants (NAD(P)H-quinone oxidoreductase, glutathione transferase, glutathione peroxidase, sirtuin-1, and thioredoxin), which are essential in ROS metabolism, the detoxification of xenobiotics, and inhibition progression of cancer cells (by stimulating apoptosis and cell cycle arrest, according to the hormesis pathways) [59,60,61]. Figure 4 represents hormesis and GB activation of NRf2, and Figure 5 shows a graphic representation for dose–response of *GB* extracts.

**Table 3:** Main bioactive compounds found in *Ginkgo biloba*.

Bioactive Compounds	Molecular Structures	Part of the Plant	Health Effects	References
Bilobalide	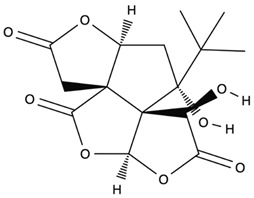	Leaves and bark.	Anti-inflammatory, antioxidant, anti-adipogenesis, pro-autophagy, and microcirculation-improving properties.	[62,63,64]
*Ginkgo*lide A	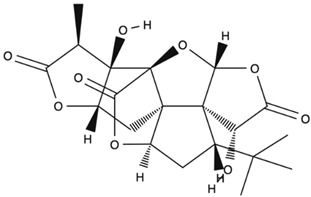	Leaves mostly, root and bark.	Anti-inflammatory, antioxidant, anxiolytic-like, anti-atherosclerosis, anti-thrombosis, neuroprotective, and hepatoprotective properties.	[65,66,67]
*Ginkgo*lide B	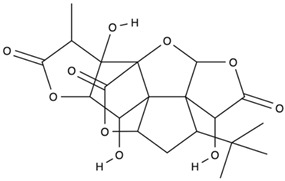	Leaves mostly, root and bark.	Anti-inflammatory, antioxidant, antiplatelet aggregation, and anti-shock properties.	[68,69,70]
*Ginkgo*lide C	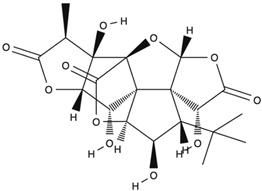	Leaves mostly, root and bark.	Anti-inflammatory, antioxidant, anticancer, and anti-adipogenesis properties.	[71,72]
Isorhamnetin	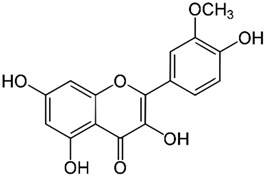	Leaves.	Anti-inflammatory, antioxidant, anti-obesity, anti-tumor, neuroprotective, cardioprotective, and organ protection properties.	[73,74,75]
Kaempferol	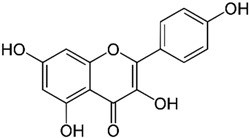	Leaves.	Anti-inflammatory, antioxidant, anticancer, cardioprotective, neuroprotective, and anticancer properties.	[76,77]
Luteolin	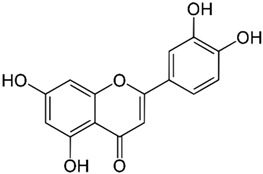	Leaves.	Anti-inflammatory, antioxidant, anticancer, neuroprotective properties.	[35,78,79,80,81]
Quercetin	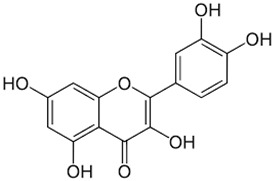	Leaves.	Anti-inflammatory, antioxidant, anticancer properties; reduces degradation of serotonin by monoamine oxidases.	[78,82,83,84,85,86,87]
Resveratrol	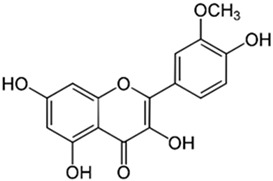	Oil, skin, roots, and leaves.	Anti-inflammatory, antioxidant, anti-obesity, antidiabetic, anti-hypertension, neuroprotective, cardioprotective, and anticancer properties.	[88,89,90,91]
Trans-arachidin-1	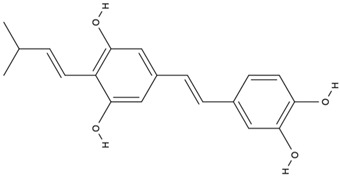	Oil, skin, roots, and leaves.	Anti-inflammatory, antioxidant, cardioprotective, and anticancer properties.	[92,93,94,95]

Tissue damage activates an inflammatory cascade, a natural response that includes complex molecular reactions and cellular responses. Lysosomal enzymes, such as phospholipase A2 (PLA2), are critical at the start of the cascade because they catalyze the hydrolysis of the sn2 position of membrane glycerophospholipids, resulting in 1-acylphospholipids and free fatty acids such as arachidonic acid. Cyclooxygenase (COX) and lipoxygenase (LOX) degrade arachidonic acid, producing prostaglandins, thromboxanes, leukotrienes, and lipoxins. These substances play a role in vasodilation, platelet aggregation, leukocyte chemotaxis, and monocyte adhesion. When activated excessively or persistently, inflammation can damage organs and systems, resulting in decompensation, organ dysfunction, and death [96,97,98,99].

Genetic links between inflammation-associated factors strengthen the link between inflammation and AD. The amyloid peptides of neuritic plaques and the tau protein of neurofibrillary tangles are alterations resulting from post-translational modifications that affect multiple genes, making AD a polygenic neurodegenerative complex disease. β-amyloid accumulation and blood–brain barrier dysfunction contribute to neuroinflammation. Furthermore, neurodegenerative diseases have been linked to neuroinflammation and viral infections, with research indicating that exposure to common viral pathogens increases the risk of conditions such as AD [100,101,102].

**Figure 6:**
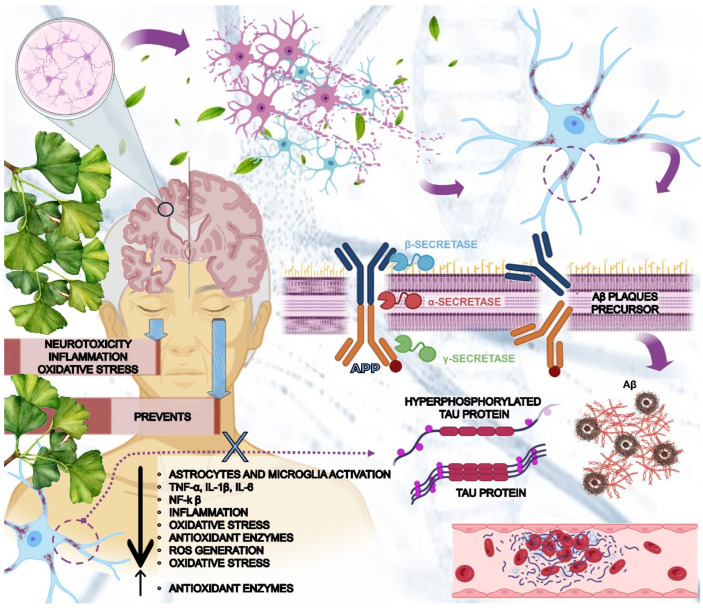
Protective mechanisms of *Ginkgo biloba* against Alzheimer’s disease (AD)*. Gingko biloba* and its extracts can play protective roles against AD and can interfere with the formation of βA plaques. Furthermore, this plant can reduce inflammatory processes by down-regulating the release of pro-inflammatory cytokines such as IL-1, IL-6, and TNF- α. Moreover, it can reduce ROS generation, oxidative stress, and apoptosis. This scenario offers prevention of AD. ↑—increase; ↓—decrease; APP—amyloid precursor protein; βA—β-amyloid; GB—*Ginkgo biloba*; IL—interleukin; NF-KB—nuclear factor KB; ROS—reactive oxygen species; TNF-α: tumor necrosis factor-α.

Their accumulation in the parenchyma and blood vessels induces microglial migration and promotes acute and chronic anti-aggregate inflammatory responses, resulting in the production of nitric oxide, reactive oxygen species (ROS), and proinflammatory cytokines such as Interleukin (IL)-1, IL-6, tumor necrosis factor-α (TNF-α), and prostaglandins (PGE2). This can eventually promote neuronal death (Figure 6) [103,104,105,106,107].

*GB* has exceptional anti-inflammatory properties due to its unique composition of bioactive compounds. GbE (standardized GB extract) inhibits the secretion of proinflammatory mediators and cytokines, such as nitric oxide, PGE2, TNF-α, IL-6, and IL-1β (Figure 6). *Ginkgo*lide A is the primary compound of GbE that can inhibit cyclooxygenase-2 (COX-2) and 5-lipoxygenase (5-LOX), preventing the production of inflammatory substances [56,108,109,110]. According to studies, GbE treatment significantly altered proteins involved in inflammation (mast cell protease-1, complement C3, T-kininogen 1) and oxidative stress (peroxiredoxin 1) (Figure 6). These proteins are related to the oxidation–reduction process, which is closely linked to inflammation [111,112]. GbE upregulates the expression of lactoylglutathione lyase (glyoxalase I), an enzyme that has anti-inflammatory properties. This enzyme is responsible for neutralizing methylglyoxal, a highly toxic compound produced during glycolysis and lipid peroxidation that can trigger inflammation [58,113]. 

*GB* inhibits the activation of nuclear factor kappa B (NF-κB), a transcription factor that activates genes that produce proinflammatory cytokines (Figure 6). This reduces the expression of these cytokines and inflammation mediators. By inhibiting NF-kB, GB also decreases the expression of adhesion molecules, which are proteins found on the surface of cells involved in the immune response and are crucial for guiding immune cells to inflamed areas. There are various types of adhesion molecules, such as intercellular adhesion molecule 1 (ICAM-1) and VCAM-1 (Vascular Cellular Adhesion Molecule 1), that are required for leukocyte adhesion to the surface of blood vessels and subsequent migration to inflammation. By decreasing the expression of these molecules, GB can reduce leukocyte adhesion and migration, resulting in a more controlled and less intense inflammatory response (Figure 6) [114,115,116,117,118,119].

*GB* has the ability to enhance the generation of nitric oxide by endothelial cells. This is attributed to its active compounds, such as flavonoids, which have the capacity to facilitate vasodilation. As a result, *GB* causes the relaxation of blood vessel walls, leading to an augmentation in blood flow. Furthermore, this plant also exhibits antiplatelet aggregation properties. These combined factors decrease the likelihood of inflammation caused by damage to the endothelium and the development of atherosclerotic plaques, thus safeguarding multiple systems in the human body. It is important to note that following an injury, *GB* strengthens blood vessel walls, reducing blood vessel permeability. This inhibits the movement of inflammatory cells into the surrounding tissues [120,121]. Another mechanism suggests that *GB* may enhance the expression of proteins such as Bcl-xL and Bcl-2. Promoting the production of these proteins increases cell survival because fewer undergo apoptosis, lowering the likelihood of inflammation being triggered (Figure 6). On the other hand, it can inhibit the activity of pro-apoptotic proteins like Bax, effectively blocking the pro-apoptotic signaling pathway [122,123].

### 4.2. Ginkgo Biloba and Antioxidant Effects

Oxidative stress is a complex pathological process that occurs in the body as a result of aging, infections, psychological stress, and exposure to radiation and chemical agents such as drugs and pollutants over a lifetime. Increased production of ROS and free radicals, which are reactive and attack various cellular components indiscriminately, is one of the damage mechanisms. They can interact with normal structures via electron donation/acceptance, hydrogen removal, addition reactions, self-annihilation, or disproportionation, resulting in irreversible cumulative injury if the endogenous antioxidant system is compromised or if there is insufficient intake of exogenous plant-derived antioxidant substances (Figure 6). Therefore, oxidative stress has been linked to the onset of a variety of medical conditions, as well as the worsening of pre-existing pathologies [28,124,125,126,127].

It is important to note that the human body is constantly producing ROS, which are unavoidable byproducts of aerobic metabolism. In aerobic eukaryotic cells, cytochrome oxidase in the electron transport chain reduces more than 90% of oxygen to water via four-electron mechanisms that do not release ROS. The remainder produces superoxide (O_2_^•−^) via the hydroperoxyl radical, while mitochondrial respiratory byproducts at 7.4 pH and NADPH oxidase catalyze the synthesis of hydrogen peroxide (H_2_O_2_). Superoxide can undergo a transformation into hydrogen peroxide. This hydrogen peroxide can then be converted into different reactive oxygen species (ROS) such as hydroxyl radicals (^•^OH) and hydroxyl anions (OH^−^). Finally, catalase facilitates the conversion of these ROS into water [128,129,130,131].

Under normal conditions, various mechanisms exist to combat free radicals. The endogenous antioxidant system consists of uncoupling protein enzymes (superoxide dismutase, glutathione peroxidase, coenzyme Q, and catalase) as well as non-enzymatic molecules that play an important role in the dissipation of free radicals. On the other hand, there is an exogenous antioxidant system that is derived from diet and plant sources such as vitamins A, C, and E, beta-carotene, lycopene, resveratrol, and flavonoids [131,132,133,134,135,136,137,138,139,140].

Regardless of how they work, their anti-aging, anti-cancer, anti-cataract, and antidiabetic properties have been demonstrated, as oxidative stress is largely responsible for disease pathophysiology. GB contains numerous compounds that are beneficial to brain health. Among them, *Ginkgo*lide A stands out for its intriguing effects on cardiovascular and cerebrovascular diseases. Its mechanism of action involves inhibiting a biomarker of oxidative stress, 8-hydroxy-2′-deoxyguanosine (8-OHdG), which is abundant in the brain following inflammation caused by, say, trauma [141]. GB’s effects on oxidative stress and neuronal protection are linked to a reduction in ROS formation and action, such as inhibiting NADPH oxidase activation, downregulating the Mitogen-Activated Protein Kinases (MAPK) and activator protein-1 (AP-1) complex, inactivating Signal Transducer and Activator of Transcription 5 (STAT5), and several other molecules (Figure 6) [142,143,144].

*Ginkgo* biloba (GB) contains bioactive compounds, including flavonoids (approximately 28%) and terpenic lactones (2.8–3.4% of *Ginkgo*lides A, B, and C, and 2.6–3.2% of bilobalide). These compounds have the ability to enhance brain circulation by reducing peroxide levels in cerebellar neurons and protecting cortical neurons from injuries caused by iron. Superoxide dismutase, catalase, and glutathione peroxidase are examples of antioxidant enzymes whose mRNA expressions are positively regulated by this plant, which reduces the generation of ROS and free radicals [145,146,147,148]. *Ginkgo*lides A, B, and C can reduce ROS production and levels (Figure 6). For example, *Ginkgo*lide A has been shown to reduce malonaldehyde production while increasing glutathione peroxidase and superoxide dismutase expression [149,150,151,152].

Kaempferol can also be found in GB, which is associated with an increase in the expression of brain-derived neurotrophic factor (BDNF), glutamate-cysteine ligase catalytic subunit, B-cell lymphoma protein 2 (BCL-2), and glutathione peroxidases. It can inhibit serotonin degradation, cytochrome C release, caspase-3 activity, and apoptosis (Figure 6). Kaempferol can reduce the neurotoxicity associated with 3-nitropropionic acid [148,153,154]. Quercetin, bilobalide, and isorhamnetin are also found in GB [74]. These phytocounphenols are related to the reduction in inflammatory processes and the production of ROS (Figure 6). Quercetin and bilobalide can work as free radical scavengers. Bilobalide is associated with the stimulation of the expression of cytochrome c oxidase subunit III and BCL-2. Isorhamnetin inhibits DNA fragmentation and apoptosis (Figure 6) [74,155,156,157].

EGB 761^®^ inhibits the formation of hydrogen peroxide radicals, anion superoxide radicals, ROS, reactive nitrogen species, peroxyl radicals, and hydroxyl radicals. Nonetheless, EGB 761^®^ plays a role in increasing the expression of glutathione peroxidase and superoxide dismutase [158,159,160]. Similarly, GB extract (GBE) has a broad pharmacological spectrum for preventing oxidative stress, which protects the central nervous system from depressive processes, improves aspects of impaired neuroplasticity, and increases cellular mitochondrial function (Figure 2 and Figure 6). Secondarily, GBE’s antioxidant and anti-inflammatory properties improve the prognosis of cardiovascular diseases [161,162,163,164].

### 4.3. Ginkgo Biloba and Alzheimer’s Disease and Dementia: Evidence from Cellular and In Vivo Studies

In vitro, *Ginkgo*lide A treatment reduced the expression of pro-inflammatory mediators like COX2 and nitric oxide, as well as cytokines like TNF-α, IL-1, and IL-1β in mouse macrophages and differentiated human monocytes [165]. Furthermore, it has been shown to significantly reduce neurological deficit scores and brain infarct volume in rats suffering from cerebral ischemia/reperfusion damage [166], as well as repair mitochondrial dysfunction in cell culture [167]. *Ginkgo*lide B has been shown to inhibit glutamate-induced apoptosis in astrocytes in vitro when glutamate metabolism is abnormal in the pathological environment of AD (Figure 6) [168]. According to studies, it improves neurological function by promoting the proliferation and differentiation of neural stem cells in rats with cerebral ischemia/reperfusion injury [169]. Electrophysiological recordings from the brain slices of rats exposed to hypoxia in vivo showed increases in the frequency of spontaneous discharge, the frequency of action potentials, and the magnitude of calcium currents. Pre-treatment with *Ginkgo*lide B, which may regulate Ca^2+^ influx in hippocampal neurons, suppressed all of these effects of hypoxia [170]. Figure 6 shows the effects of GB on AD.

In model rats with middle cerebral artery occlusion/reperfusion, *Ginkgo*lide C suppresses the CD40/NF-κB pathway to ameliorate cerebral ischemia or reperfusion-induced inflammatory impairments [165]. This compound can inhibit adipogenic factors and enzymes, increase lipolysis in a culture of differentiated adipocytes [171], and exhibit an intriguing anti-neoplastic effect in hepatocellular carcinoma cells [72], suggesting that other tissues may respond better to its application. It has been demonstrated that bilobalide increases neurogenesis and synaptogenesis in the cells of rat fetuses by stimulating the proliferation of hippocampal progenitor cells in a dose-dependent manner [172]. It supports the use of APP processing promoted by alpha-secretase in delaying the onset of AD by reducing the production of beta-amyloid in the human neuroblastoma cell line. Furthermore, studies have shown that bilobalide improves cognitive functions in AD mice [173].

In an in vitro model of AD, resveratrol can reduce oxidative damage to neurons caused by beta-amyloid via the mitophagy pathway [174]. It also had a significant effect on the integrity of the blood–brain barrier in AD-induced rats [175]. This substance was found to have good anticholinergic effects in mice with dementia when combined with other antioxidants such as vitamin E [176]. 

In a culture of human umbilical endothelial cells, kaempferol was discovered to bind to vascular endothelial growth factor, improving some angiogenic functions [112]. Kaempferol increased dopaminergic and cholinergic neurotransmission in lab rats’ prefrontal cortices, improving cognitive function [177]. It has a synergic effect on the learning and memory capabilities of model rats with AD [178]. Isorhamnetin has been shown to stimulate neurofilament production, which enhances neurite outgrowth and NGF-induced neurofilament expression.

### 4.4. Ginkgo Biloba and Neurotransmitters Related to Alzheimer’s Disease

#### 4.4.1. Acetylcholine

Several studies indicate that *GB* may have a direct and indirect impact on cholinergic function [179]. Scopolamine, a muscarinic receptor antagonist, has been shown to cause memory dysfunction in models, supporting this theory. Memory and cognitive function have been demonstrated to be negatively impacted by its administration’s transient blockade of cholinergic muscarinic receptors [180]. In rodents, *GB* treatment reduces scopolamine-induced amnesia, indicating improved cognition. This condition was associated with *GB*’s direct action on cholinergic receptors. This plant appears to directly affect presynaptic cholinergic nerve terminals, inhibiting choline absorption, which is a precursor to acetylcholine synthesis [181]. Other learning and memory models applied to rodents demonstrated the effectiveness of *GB* in memory acquisition and retention in the face of prolonged treatment [182], as well as an improvement in the animals’ memory while using a maze [183]. The study found that using *GB* in healthy young and elderly humans improved both short-term [184] and long-term [185] memory. In Alzheimer’s patients, a 3-to-6-month treatment with 120 to 240 mg of *GB* extract has a small but significant effect on *GB*’s clinical efficacy [186]. The investigation of the mechanisms involved in the effects of *GB* on cholinergic functions can bring to light new insights on the therapeutic for Alzheimer’s disease.

#### 4.4.2. Glutamate and Dopamine

*Ginkgo*lides, biologically active terpene lactones in *GB*, regulate glutamate transmission in the cortex and hippocampus. The binding of glutamate to receptors activates a short-term modulation pathway that allows ions to enter and exit the cell. The flow of calcium ions triggers the release of glutamate [187]. *Ginkgo*lides influence phospholipase A2 and prevent kinase C activation. In turn, kinase C has a significant effect on calcium circulation by influencing endoplasmic reticulum calcium release. When this pathway is inhibited, calcium flow is blocked, and glutamate cannot be released into the synapse. Thus, *Ginkgo*lides’ inhibitory function is demonstrated by their ability to reduce glutamate-induced damage in hippocampal neuronal cells in the face of cerebral ischemia [188]. Normal excitatory neurotransmission requires glutamate to be removed from the synapse by its transporters [189]. Failure to withdraw causes toxicity that can cause acute neurodegenerative diseases like epilepsy and hypoxia, as well as chronic neurodegenerative diseases like Alzheimer’s and Huntington’s Syndrome [190].

Similarly, the presence of *Ginkgo*lides appears to modulate the neurotransmitter dopamine, which regulates cognition, voluntary movements, and the activation of the punishment and reward system [191]. Chronic *Ginkgo*lide administration increases dopaminergic and noradrenergic transmission in the frontal cortex of the brain [189]. Furthermore, *Ginkgo*lides boost dopaminergic activity in rats’ paraventricular nuclei [192]. This effect can be explained by the inhibitory effect of *Ginkgo*lides on MAO (monoamine oxidase). MAO is responsible for the elimination of norepinephrine at synapses. Yoshaitatake et al. demonstrated that a single oral dose did not affect monoamine concentration levels [189]. Dysregulation of dopamine homeostasis is associated with the pathogenesis of neurodegenerative diseases. For example, growth hormone receptor 1α (GHSR1α)-induced disruption of dopamine D1 receptor (DRD1) function exacerbates the pathophysiology of AD [193].

#### 4.4.3. Serotonin—5HT

Changes in behavior, as in the case of depression, can be identified in individuals with AD [194], suggesting functional changes in the monoaminergic system, not only in the cholinergic system [195]. Some studies have shown that serotonin receptors, in addition to increasing cholinergic neurotransmission, also improve neurogenesis processes and neuronal plasticity, as well as reduce amyloid load in the brain [196,197].

In relation to serotonin 5-HT1A receptors, a decrease in their expression is observed in the aging phase [198]. However, this condition can be reversed with treatment with GB [199]. Although the mechanisms of action of GB in neuroprotective events are not well understood, the modulation of serotonin levels was observed, as well as the increase in dopamine levels due to the reduction in monoamine oxidase (MAO) activity in the prefrontal cortex in the presence of this substance [200,201,202,203].

In rats that received GB treatment for 3 weeks with an average consumption of 50–300 mg of GB, a slight increase in serotonin levels was observed in brain regions such as the prefrontal cortex and hippocampus [204].

The evaluations of the study show that 5-HT has an important role in neurodegenerative diseases, and the understanding of its mechanisms can bring new insights into AD treatment.

### 4.5. Ginkgo Biloba and miAlzheimer’s Disease and Dementia: The Results of Clinical Trials

Patients treated with MEMO (a combination of 750 mg of lyophilized royal jelly and 120 mg of standardized extracts of GB) had higher MMSE (Mini-Mental State Examination) scores than the control group (+2.07 and +0.13, respectively). The trial was randomized, double-blind, and placebo-controlled, which was a plus; however, the follow-up period was brief [34]. The protective effect of GB extract on the incidence of Alzheimer’s disease was not supported by conclusive research. The study’s sample size included over 2500 randomly assigned patients who were evaluated on a five-year cycle. Despite this, the trial failed to demonstrate a protective effect because the number of dementia events was significantly lower than expected, resulting in a lack of statistical power to detect effects. Furthermore, more than 700 patients dropped out, which was a negative point [35].

The efficacy of *GB* was evaluated, as well as its tolerability in AD. The *GB*-treated group’s MMSE scores increased from 16.52 + −4.124 to 16.76 + −4.116, with no significant difference (*p* > 0.05) [34]. *GB* enhanced cognitive functioning, neuropsychiatric symptoms, and functional abilities in dementia patients. The SKT total score (drug–placebo differences: 1.7 for AD, *p* < 0.001, and 1.4 for VaD, *p* < 0.05) and the NPI total score (drug–placebo differences: 3.1 for AD, *p* < 0.001, and 3.2 for VaD, *p* < 0.05) demonstrated a significant drug–placebo difference. The results could not be properly interpreted due to the high prevalence of mixed pathologies and the challenges associated with accurate diagnostic classification. Nevertheless, the sample selected was representative of the patients with dementia encountered in daily practice and was appropriately allocated to each type of dementia diagnosed [39]. 

A once-daily dosage of EGb 761 significantly improves patients’ neuropsychiatric symptoms and cognitive function, making it an effective treatment for dementia. On the SKT total score, patients’ improvements ranged from 2.2 to 3.5 points. There was adequate follow-up and randomization; however, the patients’ low average age and enrollment as outpatients may have limited the generalizability of the results [38].

Patients who received the *GB* extract improved by −1.4 points on the SKT and −3.2 points on the NPI total score, whereas those who received placebo deteriorated by +0.3 on the SKT and did not change on the NPI total score. *Ginkgo* outperformed placebo on all secondary outcome measures. Positive aspects of the study include the requirement for a computer tomography or magnetic resonance imaging that was no more than one year old to confirm the inclusion diagnosis and to show no evidence of other brain lesions that could account for the cognitive deficit, which increases the research’s reliability, as well as small and balanced losses between the groups at the end of the trial. The fact that there were more women than men included in the study could be a source of bias. According to the authors, a once-daily *Ginkgo* dosing regimen is both safe and effective for dementia [39]. Newly developed *GB* leaf extract is a safe, effective, and, at the very least, adjuvant treatment option for patients with mild cognitive impairment, improving symptoms of forgetfulness, impaired concentration, and impaired memory. The short duration of treatment may be a bias in this study, and the use of medications may result in an improvement in mental capacity during the study [40].

The combined treatment of *GB* plus donepezil was found to be consistently and slightly more effective than administering these medications separately. Positive aspects of the study include the recruitment of patients from a large proportion of disabled Alzheimer’s patients with behavioral and psychological symptoms, as well as the requirement of computer tomography or magnetic resonance imaging to confirm the diagnosis of probable AD. The study’s limitations include its small sample size [41].

*Ginkgo* is ineffective in preventing or delaying the onset of all-cause dementia in participants older than 75 years of age. This was demonstrated in the largest clinical trial to date evaluating the impact of *GB* on the incidence of dementia, involving over three thousand patients. The study’s strengths include the large number of patients under investigation and the balanced proportion of men and women (46% at baseline). Furthermore, the researchers included measures of overall cognitive decline and disability as secondary outcomes of the study but did not provide data in the paper, which could help us understand whether *Ginkgo* has a short-term effect on memory [42]. More evidence on the efficacy and safety of *Ginkgo* in treating cognitive and non-cognitive symptoms of dementia has been added. The treatment group improved by −3.2 points on the SKT, while the placebo group deteriorated by +1.3 points on the same test. One of the study’s major strengths was that the patients were recruited outside of the norm of daily practice, with less stringent eligibility criteria than are typically used in other dementia studies. The study’s drawback is that the small age average of 64 years may account for the patients’ high responsiveness to drug treatment, as well as the absence of death and, thus, the low rate of premature discontinuation, both situations that may distort the analysis’s results [43].

The outcomes of patients who received daily doses of *GB* extract, donepezil, or a placebo were compared. The study found statistical differences in MMSE and SKT scores between the groups, demonstrating *GB*’s effectiveness. There were no differences between the donepezil and EGb groups in this study. The study’s limitation was the small number of participants, which was insufficient for the baseline assessment [44]. Some studies on the use of GB extract in mild to moderate Alzheimer’s dementia proved inconclusive. There were no significant differences found among the groups in the entire sample. Nonetheless, patients who received the placebo experienced a minor decline in cognitive and functional aspects [205]. One study found no statistically significant differences in mean score changes between the *Ginkgo*-treated and placebo groups. There were minimal or no differences between the groups, and none of the main outcome measures were statistically significant (SKT:0.4; CGI-2:0.0; NAI-NAA:0.0). The ineffective multi-purpose design, unequal treatment distribution, and inconsistent findings with other studies are the main weaknesses of the study. The significant number of participants who left the study early is another disadvantage [48].

After EGb 761 was administered for six months, the patients in that group exhibited a small improvement. The findings from both the ITT and evaluable data show that EGb is effective in two assessment domains, specifically cognitive performance (ADAS-Cog). In terms of ADAS-Cog scores, the placebo group experienced a significant decline from their initial baseline score, whereas the EGb group showed a trend of improvement. One of the strengths of this study is its duration, which contributes to the study’s reasonable efficacy. The study suggests that more research is needed to determine the role of GB in dementia treatment and its potential as an alternative or adjunct to cholinesterase inhibitors [47].

One study found that *Ginkgo* had no effect on each of the main outcome measures for participants compared to a placebo over the course of the 24-week treatment period. Furthermore, no benefits from higher doses or longer periods of *Ginkgo* treatment were discovered. The trial results indicate that this plant is ineffective for treating older people with age-related memory impairment or mild to moderate dementia. The study’s strengths include a rigorous comparison, a comprehensive review of the existing literature, and methodological consistency. Nevertheless, the primary limitation of the study lies in its failure to establish the effectiveness of GB in enhancing cognitive and functional outcomes, thereby contradicting the prevailing body of research in this domain [48].

Due to the numerous effects of GB shown in animal models and in clinical trials, based on these findings, Table 4 was constructed to summarize these effects. 

## 5. Conclusions

This systematic review of clinical trials on the efficacy of *GB* in AD and cognitive impairment provides a nuanced perspective. While some studies showed improvements in cognitive functioning and neuropsychiatric symptoms after *GB* treatment, others found no significant differences when compared to placebo or donepezil. Notably, the combination of *GB* and donepezil provided marginal benefits, especially in patients with AD behavioral symptoms. However, large-scale trials revealed that *GB* did not effectively prevent or delay dementia onset in older people. The limitations of these studies, such as small sample sizes, inconsistent findings, and diagnostic challenges, had a significant impact on the interpretation of the results. Furthermore, some trials did not establish a protective effect of *GB* extract against AD. In light of these conflicting findings, more research with robust methodologies and larger sample sizes is needed to elucidate the role of *GB* extract in AD and cognitive impairment. Future investigations should explore optimal dosages, long-term effects, and potential synergies with existing treatments to enhance our understanding of *GB*’s therapeutic potential in dementia management.

## 6. Future Perspectives

Neuronal organoids are highly valuable tools for studying the brain because they can replicate various brain regions capable of interacting with each other or develop to resemble specific brain sections [206]. Despite variability among different brain organoids, they contain the same cell types found in the human brain. Unlike the typical layered structure of the human brain, cell subclasses in brain organoids organize into multiple layers, resembling the human brain’s development pattern [207].

The human CNS initially forms as a neural tube and later matures into distinct regions—forebrain, midbrain, and hindbrain—a process common to all mammals [208]. Cerebral organoids can mimic brain development up to 24 weeks post-conception, but afterward, they develop a necrotic core due to inadequate vascularization, impeding oxygenation and nutrient diffusion [209]. To address this challenge, researchers use spinning bioreactors to support vascularization and develop region-specific brain organoids [210]. Brain-derived neurotrophic factor (BDNF) is also used to promote neuronal survival and maturation [211]. Alternative methods include transplanting organoids into adult mouse brains or slicing organoids for orbital shaking to enhance survival and prevent necrosis [212].

Conventional two-dimensional (2D) cell culture and animal models have long been utilized in AD research to investigate AD pathology and explore potential treatments [213]. While these models have yielded valuable insights, they only capture a portion of AD mechanisms because they cannot replicate the complex tissue structure, function, and cellular diversity specific to the human brain. The development of three-dimensional (3D) cerebral organoids through tissue engineering and pluripotente-induced stem cell technology represents a recent advancement that enables the creation of models more closely resembling human brain tissue features than previous models [214].

Recent advancements in biomaterials, microfabrication, microfluidics, and cell biology have also led to the creation of organ-on-a-chip devices capable of replicating key functions of various organs. These platforms offer the potential to provide novel insights into diverse physiological processes, including disease mechanisms, and to assess the impacts of external interventions such as drug treatments [215]. While animal models remain in use, their relevance to human physiology is uncertain, and their utilization is labor-intensive and raises ethical concerns. Organ-on-a-chip systems have been developed to simulate various brain tissue components, including specific brain regions with distinct functions and the blood–brain barrier, under both normal and pathophysiological conditions.

Within this scenario, Park et al. [215] developed a novel microfluidic chip utilizing three-dimensional (3D) neurospheroids to closely replicate the brain’s in vivo microenvironment. This innovative chip facilitated a constant flow of fluid, simulating the fluid dynamics observed within the brain’s interstitial space. Additionally, the study investigated the toxic effects of amyloid-β, a key factor in AD pathogenesis. Utilizing the microfluidic chip’s osmotic micropump system, amyloid-β treatment significantly reduced neurospheroid viability and caused more extensive destruction of neural networks compared to treatment under static conditions. These findings underscore the importance of incorporating in vivo-like microenvironments to better model neurodegenerative diseases and facilitate high-throughput drug screening. Therefore, the developed 3D culture-based microfluidic chip represents a promising in vitro brain model, offering enhanced physiological relevance for studying neurodegenerative diseases and evaluating potential therapeutic interventions.

## Figures and Tables

**Table 4:** Summary of neuroprotective effects of *Ginkgo biloba* interventions in experimental models and clinical trials.

Topic	Evidence/Findings	Mechanism of Action
*Ginkgo biloba*’s Impact on Inflammatory Responses	*Ginkgo*lide A decreases the levels of pro-inflammatory mediators (such as COX2 and nitric oxide) and cytokines (including TNF-α, IL-1, and IL-1β) in cultured mouse macrophages and human monocytes. Additionally, it mitigates neurological impairments and reduces brain infarct size in rats subjected to cerebral ischemia/reperfusion injury. Meanwhile, *Ginkgo*lide B counteracts glutamate-induced cell death in astrocytes and fosters the proliferation and differentiation of neural stem cells in rats experiencing cerebral ischemia/reperfusion damage.	*Ginkgo*lide A demonstrates inhibition of pro-inflammatory mediators and cytokines, which helps to reduce inflammatory responses in various contexts. On the other hand, *Ginkgo*lide B provides protection against glutamate-induced apoptosis, offering a shield against cellular damage, and supports neural stem cell activity, which can aid in brain repair and regeneration.
*Ginkgo biloba* and Resveratrol: Potential Roles in Alzheimer’s Disease	Resveratrol mitigates neuronal oxidative damage induced by beta-amyloid and enhances the integrity of the blood–brain barrier in rats with Alzheimer’s disease.	*Ginkgo* and resveratrol function by reducing oxidative stress within neurons and enhancing the integrity of the blood–brain barrier.
*Ginkgo biloba* and Kaempferol: Exploring Their Potential in Alzheimer’s Disease	Kaempferol exhibits the ability to bind to vascular endothelial growth factor (VEGF), thereby enhancing angiogenic processes. Additionally, it enhances dopaminergic and cholinergic neurotransmission within the prefrontal cortex of rats, which leads to improved cognitive function and learning in rat models of Alzheimer’s disease.	*Ginkgo* and kaempferol contribute to improving angiogenic functions and neurotransmission in the brain.
*Ginkgo biloba* and Neurotransmitter Modulation in Alzheimer’s Disease	*Ginkgo*lides play a role in regulating both glutamate and dopamine transmission in the brain. They have been observed to modulate glutamate-induced damage and promote increased dopaminergic and noradrenergic transmission. Additionally, *Ginkgo biloba* affects serotonin levels and the expression of serotonin receptors, which may help counteract age-related declines in serotonin receptor expression.	*Ginkgo*lides modulate neurotransmission of glutamate, dopamine, and serotonin in the brain.
Exploring *Ginkgo biloba*’s Clinical Trials for Alzheimer’s Disease and Dementia	Clinical trials evaluating *Ginkgo biloba* extract (specifically EGb 761) have yielded mixed results. Some trials indicate improvements in cognitive function and reductions in neuropsychiatric symptoms when using this extract. However, other studies have not shown significant benefits in terms of preventing or delaying the onset of dementia in older adults. Interestingly, combining *Ginkgo* with donepezil (a medication used to treat Alzheimer’s disease) may result in slightly better efficacy compared to using either treatment alone. This suggests a potential synergistic effect when these treatments are used together for managing cognitive decline.	EGb 761 (*Ginkgo biloba* extract) has been associated with improvements in cognitive function and reductions in neuropsychiatric symptoms. When combined with donepezil, this treatment combination may exhibit synergistic effects, potentially enhancing therapeutic outcomes.

## Data Availability

All of the data is contained within the article.

## Abbreviations

Aβ42, amyloid-β42; ACTH, corticotrophic hormone; ADAS-Cog, Alzheimer’s disease assessment scale–cognitive subscale; ADHD, attention-deficit hyperactivity disorder; AMPA, α-amino-3-hydroxy-5-methyl-4-isoxazolepropionic acid; AQP, aquaporin; ASD, autism spectrum disorder; AVLT, Rey Auditory Verbal Learning Test; BACE, beta-site amyloid precursor protein-cleaving enzyme; Bax, Bcl-2-associated X protein; Bcl, B-cell lymphoma; BDNF, Brain-derived neurotrophic factor; BM, *Bacopa monnieri* L.; BME, *Bacopa monnieri* ethanolic extract; CAT, catalase; CCR, C-C chemokine receptor; ChAT, Choline Acetyltransferase; CNS, Central Nervous System; COX, cyclooxygenase; CREB, Cyclic AMP response element-binding protein; DAMP, damage-associated molecular patterns; DCX, Doublecortin neuronal marker; DM2, type 2 diabetes mellitus; DSM-IV, Diagnostic and Statistical Manual of Mental Disorders; ERK, extra-cellular signal-regulated kinase; FADD, FAZ-associated death domain protein; GCLC, glutamate cysteine ligase catalytic subunit; GDS, geriatric depression scale; GPx, Glutathione peroxidase; GSH, Glutathione; GSK-3B, glycogen synthase kinase; GST, glutathione S-transferase; GWI, Gulf War Illness; HO, heme oxygenase; ICF, chronic liver failure; IKK, inhibitor of nuclear factor-κB kinase; IL, Interleukin; INF- γ, Interferon-γ; iNOS, inducible Nitric Oxide synthase; JNK, c-Jun N-terminal kinases; LOX, Lipoxygenase; LPS, lipopolysaccharide; MAO, mono-amino oxidase; MARK, Microtubule affinity-regulating kinase; MCP, Monocyte chemoattractant protein; MDA, Malonaldehyde; MMP, Matrix Metalloproteinase; MMSE, Mini-Mental State Examination; MoCA, Montreal Cognitive Assessment; MPTP, 1-methyl-4-phenyl-1,2,3,6 tetrahydropyridine; MTF, multitasking framework; NF-κB, Nuclear Factor-κB; NMDAR, N-methyl-D-aspartate receptors; nNOS, neuronal nitric oxide synthase; NO, Nitric Oxide; 3-NPA, 3-nitropropionic acid; Nrf2, Nuclear factor erythroid 2-related factor 2; OGD, oxygen and glucose deprivation; OS, oxidative stress; PGES, Prostaglandin; PGIMS, Postgraduate Institute Memory Scale; PGQL, Parkinson’s Disease Quality-of-Life; PPI, protein–protein interactions; PSQ, Perceived Stress Questionnaire; PTUPB, dual inhibitor of COX and epoxide hydrolase; RNS, reactive nitrogen species; ROS, reactive oxygen species; SDAT, Senile Dementia of Alzheimer’s Type; SDQ, strength and difficulties questionnaire; SHAPS, Snaith–Hamilton Pleasure Scale; sHE, soluble Epoxide Hydrolase; SOD, Superoxide Dismutase; STAT, signal transducers and activators of transcription; TAA, thioacetamide; TNF-α, tumor necrosis factor alpha; TRADD, TNFR1-associated death domain protein; WS, *Withania somnifera* (L.) Dunal.

## References

[1] Alzheimer A. (1907). Uber eigenartige Erkrankung der Hirnrinde. Allg. Z. fur Psychiatr. und Psych.-Gerichtl. Med..

[2] Scheyer O., Rahman A., Hristov H., Berkowitz C., Isaacson R.S., Diaz Brinton R., Mosconi L. (2018). Female Sex and Alzheimer’s Risk: The Menopause Connection. J. Prev. Alzheimers Dis..

[3] Raz L., Knoefel J., Bhaskar K. (2016). The neuropathology and cerebrovascular mechanisms of dementia. J. Cereb. Blood Flow Metab..

[4] Bondi M.W., Edmonds E.C., Salmon D.P. (2017). Alzheimer’s Disease: Past, Present, and Future. J. Int. Neuropsychol. Soc..

[5] Battaglia S., Avenanti A., Vécsei L., Tanaka M. (2024). Neural Correlates and Molecular Mechanisms of Memory and Learning. Int. J. Mol. Sci..

[6] Tanaka M., Szabó Á., Körtési T., Szok D., Tajti J., Vécsei L. (2023). From CGRP to PACAP, VIP, and Beyond: Unraveling the Next Chapters in Migraine Treatment. Cells.

[7] Tanaka M., Chen C. (2023). Editorial: Towards a mechanistic understanding of depression, anxiety, and their comorbidity: Perspectives from cognitive neuroscience. Front. Behav. Neurosci..

[8] Lei P., Ayton S., Bush A.I. (2021). The essential elements of Alzheimer’s disease. J. Biol. Chem..

[9] Balestrieri J.V.L., Nonato M.B., Gheler L., Prandini M.N. (2020). Structural Volume of Hippocampus and Alzheimer’s Disease. Rev. Assoc. Med. Bras. (1992).

[10] Makowski L. (2020). The Structural Basis of Amyloid Strains in Alzheimer’s Disease. ACS Biomater. Sci. Eng..

[11] Ashrafian H., Zadeh E.H., Khan R.H. (2021). Review on Alzheimer’s disease: Inhibition of amyloid beta and tau tangle formation. Int. J. Biol. Macromol..

[12] Fukumori A., Feilen L.P., Steiner H. (2020). Substrate recruitment by γ-secretase. Semin. Cell Dev. Biol..

[13] Hur J.Y. (2022). γ-Secretase in Alzheimer’s disease. Exp. Mol. Med..

[14] Wang S., Mustafa M., Yuede C.M., Salazar S.V., Kong P., Long H., Ward M., Siddiqui O., Paul R., Gilfillan S. (2020). Anti-human TREM2 induces microglia proliferation and reduces pathology in an Alzheimer’s disease model. J. Exp. Med..

[15] Sinsky J., Pichlerova K., Hanes J. (2021). Tau Protein Interaction Partners and Their Roles in Alzheimer’s Disease and Other Tauopathies. Int. J. Mol. Sci..

[16] Lane C.A., Hardy J., Schott J.M. (2018). Alzheimer’s disease. Eur. J. Neurol..

[17] Breijyeh Z., Karaman R. (2020). Comprehensive Review on Alzheimer’s Disease: Causes and Treatment. Molecules.

[18] Van Bulck M., Sierra-Magro A., Alarcon-Gil J., Perez-Castillo A., Morales-Garcia J.A. (2019). Novel Approaches for the Treatment of Alzheimer’s and Parkinson’s Disease. Int. J. Mol. Sci..

[19] Martos D., Lőrinczi B., Szatmári I., Vécsei L., Tanaka M. (2024). The Impact of C-3 Side Chain Modifications on Kynurenic Acid: A Behavioral Analysis of Its Analogs in the Motor Domain. Int. J. Mol. Sci..

[20] Battaglia S., Schmidt A., Hassel S., Tanaka M. (2023). Editorial: Case reports in neuroimaging and stimulation. Front. Psychiatry.

[21] Martos D., Tuka B., Tanaka M., Vécsei L., Telegdy G. (2022). Memory Enhancement with Kynurenic Acid and Its Mechanisms in Neurotransmission. Biomedicines.

[22] Battaglia S., Di Fazio C., Mazzà M., Tamietto M., Avenanti A. (2024). Targeting Human Glucocorticoid Receptors in Fear Learning: A Multiscale Integrated Approach to Study Functional Connectivity. Int. J. Mol. Sci..

[23] Di Gregorio F., Battaglia S. (2023). Advances in EEG-based functional connectivity approaches to the study of the central nervous system in health and disease. Adv. Clin. Exp. Med. Off. Organ. Wroc. Med. Univ..

[24] Battaglia S., Avenanti A., Vécsei L., Tanaka M. (2024). Neurodegeneration in Cognitive Impairment and Mood Disorders for Experimental, Clinical and Translational Neuropsychiatry. Biomedicines.

[25] Cummings J. (2023). Anti-Amyloid Monoclonal Antibodies are Transformative Treatments that Redefine Alzheimer’s Disease Therapeutics. Drugs.

[26] Valotto Neto L.J., Reverete de Araujo M., Moretti Junior R.C., Mendes Machado N., Joshi R.K., dos Santos Buglio D., Barbalho Lamas C., Direito R., Fornari Laurindo L., Tanaka M.J.A. (2024). Investigating the Neuroprotective and Cognitive-Enhancing Effects of *Bacopa monnieri*: A Systematic Review Focused on Inflammation, Oxidative Stress, Mitochondrial Dysfunction, and Apoptosis. Antioxidants.

[27] Singh S.K., Srivastav S., Castellani R.J., Plascencia-Villa G., Perry G. (2019). Neuroprotective and Antioxidant Effect of *Ginkgo biloba* Extract Against AD and Other Neurological Disorders. Neurotherapeutics.

[28] Barbalho S.M., Direito R., Laurindo L.F., Marton L.T., Guiguer E.L., Goulart R.A., Tofano R.J., Carvalho A.C.A., Flato U.A.P., Capelluppi Tofano V.A. (2022). *Ginkgo biloba* in the Aging Process: A Narrative Review. Antioxidants.

[29] Aminifard T., Razavi B.M., Hosseinzadeh H. (2021). The effects of ginseng on the metabolic syndrome: An updated review. Food Sci. Nutr..

[30] Lopez O.L., Chang Y., Ives D.G., Snitz B.E., Fitzpatrick A.L., Carlson M.C., Rapp S.R., Williamson J.D., Tracy R.P., DeKosky S.T. (2019). Blood amyloid levels and risk of dementia in the Ginkgo Evaluation of Memory Study (GEMS): A longitudinal analysis. Alzheimer’s Dement. J. Alzheimer’s Assoc..

[31] Page M.J., McKenzie J.E., Bossuyt P.M., Boutron I., Hoffmann T.C., Mulrow C.D., Shamseer L., Tetzlaff J.M., Akl E.A., Brennan S.E. (2021). The PRISMA 2020 statement: An updated guideline for reporting systematic reviews. BMJ (Clin. Res. Ed.).

[32] Moher D., Liberati A., Tetzlaff J., Altman D.G., The PRISMA Group (2009). Preferred reporting items for systematic reviews and meta-analyses: The PRISMA statement. Ann. Intern. Med..

[33] Cumpston M., Li T., Page M.J., Chandler J., Welch V.A., Higgins J.P., Thomas J. (2019). Updated guidance for trusted systematic reviews: A new edition of the Cochrane Handbook for Systematic Reviews of Interventions. Cochrane Database Syst. Rev..

[34] Yakoot M., Salem A., Helmy S. (2013). Effect of Memo^®^, a natural formula combination, on Mini-Mental State Examination scores in patients with mild cognitive impairment. Clin. Interv. Aging.

[35] Vellas B., Coley N., Ousset P.-J., Berrut G., Dartigues J.-F., Dubois B., Grandjean H., Pasquier F., Piette F., Robert P. (2012). Long-term use of standardised *Ginkgo biloba* extract for the prevention of Alzheimer’s disease (GuidAge): A randomised placebo-controlled trial. Lancet Neurol..

[36] Nasab N.M., Bahrammi M.A., Nikpour M.R., Rahim F., Naghibis S.N. (2012). Efficacy of rivastigmine in comparison to ginkgo for treating Alzheimer’s dementia. JPMA J. Pak. Med. Assoc..

[37] Ihl R., Tribanek M., Bachinskaya N., for the GOTADAY Study Group (2012). Efficacy and tolerability of a once daily formulation of *Ginkgo biloba* extract EGb 761^®^ in Alzheimer’s disease and vascular dementia: Results from a randomised controlled trial. Pharmacopsychiatry.

[38] Herrschaft H., Nacu A., Likhachev S., Sholomov I., Hoerr R., Schlaefke S. (2012). *Ginkgo biloba* extract EGb 761^®^ in dementia with neuropsychiatric features: A randomised, placebo-controlled trial to confirm the efficacy and safety of a daily dose of 240 mg. J. Psychiatr. Res..

[39] Ihl R., Bachinskaya N., Korczyn A.D., Vakhapova V., Tribanek M., Hoerr R., Napryeyenko O., Group G.S. (2011). Efficacy and safety of a once-daily formulation of *Ginkgo biloba* extract EGb 761 in dementia with neuropsychiatric features: A randomized controlled trial. Int. J. Geriatr. Psychiatry.

[40] Bäurle P., Suter A., Wormstall H. (2009). Safety and effectiveness of a traditional ginkgo fresh plant extract—Results from a clinical trial. Forsch Komplementmed.

[41] Yancheva S., Ihl R., Nikolova G., Panayotov P., Schlaefke S., Hoerr R. (2009). *Ginkgo biloba* extract EGb 761(R), donepezil or both combined in the treatment of Alzheimer’s disease with neuropsychiatric features: A randomised, double-blind, exploratory trial. Aging Ment. Health.

[42] DeKosky S.T., Williamson J.D., Fitzpatrick A.L., Kronmal R.A., Ives D.G., Saxton J.A., Lopez O.L., Burke G., Carlson M.C., Fried L.P. (2008). *Ginkgo biloba* for prevention of dementia: A randomized controlled trial. JAMA.

[43] Napryeyenko O., Borzenko I. (2007). *Ginkgo biloba* special extract in dementia with neuropsychiatric features. A randomised, placebo-controlled, double-blind clinical trial. Arzneimittelforschung.

[44] Mazza M., Capuano A., Bria P., Mazza S. (2006). *Ginkgo biloba* and donepezil: A comparison in the treatment of Alzheimer’s dementia in a randomized placebo-controlled double-blind study. Eur. J. Neurol..

[45] Zhu Q.-X., Shen T., Tu D.-Y., Ding R., Liang Z.-Z., Zhang X.-J. (2005). Protective effects of *Ginkgo biloba* leaf extracts on trichloroethylene-induced human keratinocyte cytotoxicity and apoptosis. Ski Pharmacol. Physiol..

[46] Van Dongen M., van Rossum E., Kessels A., Sielhorst H., Knipschild P. (2003). Ginkgo for elderly people with dementia and age-associated memory impairment: A randomized clinical trial. J. Clin. Epidemiol..

[47] Le Bars P.L., Kieser M., Itil K.Z. (2000). A 26-week analysis of a double-blind, placebo-controlled trial of the ginkgo biloba extract EGb 761 in dementia. Dement. Geriatr. Cogn. Disord..

[48] Van Dongen M.C., van Rossum E., Kessels A.G., Sielhorst H.J., Knipschild P.G. (2000). The efficacy of ginkgo for elderly people with dementia and age-associated memory impairment: New results of a randomized clinical trial. J. Am. Geriatr. Soc..

[49] Zheng H., Wang B., Hua X., Gao R., Wang Y., Zhang Z., Zhang Y., Mei J., Huang Y., Huang Y. (2023). A near-complete genome assembly of the allotetrapolyploid *Cenchrus fungigraminus* (JUJUNCAO) provides insights into its evolution and C4 photosynthesis. Plant. Commun..

[50] Singh B., Kaur P., Singh R., Ahuja P. (2008). Biology and chemistry of *Ginkgo biloba*. Fitoterapia.

[51] Chang B., Qiu X., Yang Y., Zhou W., Jin B., Wang L. (2024). Genome-wide analyses of the GbAP2 subfamily reveal the function of GbTOE1a in salt and drought stress tolerance in *Ginkgo biloba*. Plant Sci. Int. J. Exp. Plant Biol..

[52] Gertz H.J., Kiefer M. (2004). Review about *Ginkgo biloba* special extract EGb 761 (Ginkgo). Curr. Pharm. Des..

[53] Sun Y., Bai P.P., Gu K.J., Yang S.Z., Lin H.Y., Shi C.G., Zhao Y.P. (2022). Dynamic transcriptome and network-based analysis of yellow leaf mutant *Ginkgo biloba*. BMC Plant. Biol..

[54] Krauze-Baranowska M., Sowiński P. (1999). 2,3-Dihydrobiflavone from *Ginkgo biloba*. Planta Medica.

[55] Gregory J., Vengalasetti Y.V., Bredesen D.E., Rao R.V. (2021). Neuroprotective Herbs for the Management of Alzheimer’s Disease. Biomolecules.

[56] Zhang S., Gong X., Qu H. (2024). An effective and comprehensive optimization strategy for preparing *Ginkgo biloba* leaf extract enriched in shikimic acid by macroporous resin column chromatography. Phytochem. Anal. PCA.

[57] Rong Y., Geng Z., Lau B.H. (1996). *Ginkgo biloba* attenuates oxidative stress in macrophages and endothelial cells. Free Radic. Biol. Med..

[58] Hirata B.K.S., Pedroso A.P., Machado M.M.F., Neto N.I.P., Perestrelo B.O., de Sá R., Alonso-Vale M.I.C., Nogueira F.N., Oyama L.M., Ribeiro E.B. (2019). *Ginkgo biloba* Extract Modulates the Retroperitoneal Fat Depot Proteome and Reduces Oxidative Stress in Diet-Induced Obese Rats. Front. Pharmacol..

[59] Calabrese E.J., Calabrese V., Tsatsakis A., Giordano J.J. (2020). Hormesis and *Ginkgo biloba* (GB): Numerous biological effects of GB are mediated via hormesis. Ageing Res. Rev..

[60] Amara I., Salah A., Timoumi R., Annabi E., Scuto M., Trovato A., Neffati F., Calabrese V., Abid-Essefi S. (2020). Effect of di(2-ethylhexyl) phthalate on Nrf2-regulated glutathione homeostasis in mouse kidney. Cell Stress Chaperones.

[61] Scuto M., Ontario M.L., Salinaro A.T., Caligiuri I., Rampulla F., Zimbone V., Modafferi S., Rizzolio F., Canzonieri V., Calabrese E.J. (2022). Redox modulation by plant polyphenols targeting vitagenes for chemoprevention and therapy: Relevance to novel anti-cancer interventions and mini-brain organoid technology. Free Radic. Biol. Med..

[62] Qin Y.R., Ma C.Q., Wang D.P., Zhang Q.Q., Liu M.R., Zhao H.R., Jiang J.H., Fang Q. (2021). Bilobalide alleviates neuroinflammation and promotes autophagy in Alzheimer’s disease by upregulating lincRNA-p21. Am. J. Transl. Res..

[63] Lu J., Xie L., Liu K., Zhang X., Wang X., Dai X., Liang Y., Cao Y., Li X. (2021). Bilobalide: A review of its pharmacology, pharmacokinetics, toxicity, and safety. Phytother. Res. PTR.

[64] Bu S., Yuan C.Y., Xue Q., Chen Y., Cao F. (2019). Bilobalide Suppresses Adipogenesis in 3T3-L1 Adipocytes via the AMPK Signaling Pathway. Molecules.

[65] Wang L., Zhao Y., Su Z., Zhao K., Li P., Xu T. (2023). Ginkgolide A targets forkhead box O1 to protect against lipopolysaccharide-induced septic cardiomyopathy. Phytother. Res. PTR.

[66] Zhao K., Li Y., Zhou Z., Mao Y., Wu X., Hua D., Yong Y., Li P. (2022). Ginkgolide A alleviates cardiac remodeling in mice with myocardial infarction via binding to matrix metalloproteinase-9 to attenuate inflammation. Eur. J. Pharmacol..

[67] He H., Ge J., Yi S., Wang Y., Liu Y., Liu Y., Liu X. (2023). Ginkgolide A downregulates transient receptor potential (melastatin) 2 to protect cisplatin-induced acute kidney injury in rats through the TWEAK/Fn14 pathway: Ginkgolide A improve acute renal injury. Hum. Exp. Toxicol..

[68] Chen J., Ou Z., Gao T., Yang Y., Shu A., Xu H., Chen Y., Lv Z. (2022). Ginkgolide B alleviates oxidative stress and ferroptosis by inhibiting GPX4 ubiquitination to improve diabetic nephropathy. Biomed. Pharmacother..

[69] Yang Y., Wu Q., Shan X., Zhou H., Wang J., Hu Y., Chen J., Lv Z. (2024). Ginkgolide B attenuates cerebral ischemia-reperfusion injury via inhibition of ferroptosis through disrupting NCOA4-FTH1 interaction. J. Ethnopharmacol..

[70] Wang Q., Ni S., Ling L., Wang S., Xie H., Ren Z. (2023). Ginkgolide B Blocks Vascular Remodeling after Vascular Injury via Regulating Tgf β 1/Smad Signaling Pathway. Cardiovasc. Ther..

[71] Huang Y., Zhu D., Ciais P., Guenet B., Huang Y., Goll D.S., Guimberteau M., Jornet-Puig A., Lu X., Luo Y. (2018). Matrix-Based Sensitivity Assessment of Soil Organic Carbon Storage: A Case Study from the ORCHIDEE-MICT Model. J. Adv. Model. Earth Syst..

[72] Yang M.H., Baek S.H., Um J.-Y., Ahn K.S.J.I. (2020). Anti-neoplastic effect of ginkgolide C through modulating c-met phosphorylation in hepatocellular carcinoma cells. Int. J. Mol. Sci..

[73] González-Arceo M., Gomez-Lopez I., Carr-Ugarte H., Eseberri I., González M., Cano M.P., Portillo M.P., Gómez-Zorita S. (2022). Anti-Obesity Effects of Isorhamnetin and Isorhamnetin Conjugates. Int. J. Mol. Sci..

[74] Gong G., Guan Y.Y., Zhang Z.L., Rahman K., Wang S.J., Zhou S., Luan X., Zhang H. (2020). Isorhamnetin: A review of pharmacological effects. Biomed. Pharmacother..

[75] Xu S.L., Choi R.C.Y., Zhu K.Y., Leung K.-W., Guo A.J.Y., Bi D., Xu H., Lau D.T.W., Dong T.T.X., Tsim K.W.K. (2012). Isorhamnetin, A Flavonol Aglycone from *Ginkgo biloba* L., Induces Neuronal Differentiation of Cultured PC12 Cells: Potentiating the Effect of Nerve Growth Factor. Evid.-Based Complement. Altern. Med. Ecam.

[76] Periferakis A., Periferakis K., Badarau I.A., Petran E.M., Popa D.C., Caruntu A., Costache R.S., Scheau C., Caruntu C., Costache D.O. (2022). Kaempferol: Antimicrobial Properties, Sources, Clinical, and Traditional Applications. Int. J. Mol. Sci..

[77] Qattan M.Y., Khan M.I., Alharbi S.H., Verma A.K., Al-Saeed F.A., Abduallah A.M., Al Areefy A.A. (2022). Therapeutic Importance of Kaempferol in the Treatment of Cancer through the Modulation of Cell Signalling Pathways. Molecules.

[78] Belwal T., Giri L., Bahukhandi A., Tariq M., Kewlani P., Bhatt I.D., Rawal R.S., Nabavi S.M., Silva A.S. (2019). *Ginkgo* *biloba*. Nonvitamin and Nonmineral Nutritional Supplements.

[79] Shukla R., Pandey V., Vadnere G.P., Lodhi S., Watson R.R., Preedy V.R. (2019). Role of Flavonoids in Management of Inflammatory Disorders. Bioactive Food as Dietary Interventions for Arthritis and Related Inflammatory Diseases.

[80] Sathya S., Pandima Devi K., Farooqui T., Farooqui A.A. (2018). The Use of Polyphenols for the Treatment of Alzheimer’s Disease. Role of the Mediterranean Diet in the Brain and Neurodegenerative Diseases.

[81] Luo Y., Shang P., Li D. (2017). Luteolin: A Flavonoid that Has Multiple Cardio-Protective Effects and Its Molecular Mechanisms. Front. Pharmacol..

[82] Milanezi F.G., Meireles L.M., de Christo Scherer M.M., de Oliveira J.P., da Silva A.R., de Araujo M.L., Endringer D.C., Fronza M., Guimarães M.C.C., Scherer R. (2019). Antioxidant, antimicrobial and cytotoxic activities of gold nanoparticles capped with quercetin. Saudi Pharm. J..

[83] Ferenczyova K., Kalocayova B., Bartekova M. (2020). Potential Implications of Quercetin and its Derivatives in Cardioprotection. Int. J. Mol. Sci..

[84] Almatroodi S.A., Alsahli M.A., Almatroudi A., Verma A.K., Aloliqi A., Allemailem K.S., Khan A.A., Rahmani A.H. (2021). Potential Therapeutic Targets of Quercetin, a Plant Flavonol, and Its Role in the Therapy of Various Types of Cancer through the Modulation of Various Cell Signaling Pathways. Molecules.

[85] Li H., Xiao L., He H., Zeng H., Liu J., Jiang C., Mei G., Yu J., Chen H., Yao P. (2021). Quercetin Attenuates Atherosclerotic Inflammation by Inhibiting Galectin-3-NLRP3 Signaling Pathway. Mol. Nutr. Food Res..

[86] Boots A.W., Drent M., de Boer V.C.J., Bast A., Haenen G.R.M.M. (2011). Quercetin reduces markers of oxidative stress and inflammation in sarcoidosis. Clin. Nutr..

[87] Chen S., Jiang H., Wu X., Fang J. (2016). Therapeutic Effects of Quercetin on Inflammation, Obesity, and Type 2 Diabetes. Mediat. Inflamm..

[88] Kris-Etherton P.M., Hu F.B., Ros E., Sabaté J. (2008). The role of tree nuts and peanuts in the prevention of coronary heart disease: Multiple potential mechanisms. J. Nutr..

[89] Park S.H., Do M.H., Lee J.H., Jeong M., Lim O.K., Kim S.Y. (2017). Inhibitory Effect of *Arachis hypogaea* (Peanut) and Its Phenolics against Methylglyoxal-Derived Advanced Glycation End Product Toxicity. Nutrients.

[90] Raghu S.V., Kudva A.K., Krishnamurthy R.G., Mudgal J., George T., Baliga M.S. (2023). Neuroprotective effects of dietary plants and phytochemicals against radiation-induced cognitive and behavioral deficits: A comprehensive review of evidence and prospects for future research. Food Funct..

[91] Cavalcante de Freitas P.G., Rodrigues Arruda B., Araújo Mendes M.G., Barroso de Freitas J.V., da Silva M.E., Sampaio T.L., Petrilli R., Eloy J.O. (2023). Resveratrol-Loaded Polymeric Nanoparticles: The Effects of D-α-Tocopheryl Polyethylene Glycol 1000 Succinate (TPGS) on Physicochemical and Biological Properties against Breast Cancer In Vitro and In Vivo. Cancers.

[92] Mingrou L., Guo S., Ho C.T., Bai N. (2022). Review on chemical compositions and biological activities of peanut (*Arachis hypogeae* L.). J. Food Biochem..

[93] Eungsuwan N., Chayjarung P., Pankam J., Pilaisangsuree V., Wongshaya P., Kongbangkerd A., Sriphannam C., Limmongkon A. (2021). Production and antimicrobial activity of trans-resveratrol, trans-arachidin-1 and trans-arachidin-3 from elicited peanut hairy root cultures in shake flasks compared with bioreactors. J. Biotechnol..

[94] Abbott J.A., Medina-Bolivar F., Martin E.M., Engelberth A.S., Villagarcia H., Clausen E.C., Carrier D.J. (2010). Purification of resveratrol, arachidin-1, and arachidin-3 from hairy root cultures of peanut (*Arachis hypogaea*) and determination of their antioxidant activity and cytotoxicity. Biotechnol. Prog..

[95] Condori J., Sivakumar G., Hubstenberger J., Dolan M.C., Sobolev V.S., Medina-Bolivar F.J. (2010). Induced biosynthesis of resveratrol and the prenylated stilbenoids arachidin-1 and arachidin-3 in hairy root cultures of peanut: Effects of culture medium and growth stage. Lant Physiol. Biochem..

[96] Medzhitov R. (2008). Origin and physiological roles of inflammation. Nature.

[97] Mack M. (2018). Inflammation and fibrosis. Matrix Biol..

[98] Liu C., Chu D., Kalantar-Zadeh K., George J., Young H.A., Liu G. (2021). Cytokines: From Clinical Significance to Quantification. Adv. Sci..

[99] Holmes C. (2013). Review: Systemic inflammation and Alzheimer’s disease. Neuropathol. Appl. Neurobiol..

[100] Battaglia S., Nazzi C., Thayer J.F. (2024). Genetic differences associated with dopamine and serotonin release mediate fear-induced bradycardia in the human brain. Transl. Psychiatry.

[101] Di Gregorio F., Steinhauser M., Maier M.E., Thayer J.F., Battaglia S. (2024). Error-related cardiac deceleration: Functional interplay between error-related brain activity and autonomic nervous system in performance monitoring. Neurosci. Biobehav. Rev..

[102] Battaglia S., Nazzi C., Thayer J.F. (2023). Heart’s tale of trauma: Fear-conditioned heart rate changes in post-traumatic stress disorder. Acta Psychiatr. Scand..

[103] Heneka M.T., Carson M.J., El Khoury J., Landreth G.E., Brosseron F., Feinstein D.L., Jacobs A.H., Wyss-Coray T., Vitorica J., Ransohoff R.M. (2015). Neuroinflammation in Alzheimer’s disease. Lancet Neurol..

[104] Naseri N.N., Wang H., Guo J., Sharma M., Luo W. (2019). The complexity of tau in Alzheimer’s disease. Neurosci. Lett..

[105] Calsolaro V., Edison P. (2016). Neuroinflammation in Alzheimer’s disease: Current evidence and future directions. Alzheimers Dement.

[106] Akiyama H., Barger S., Barnum S., Bradt B., Bauer J., Cole G.M., Cooper N.R., Eikelenboom P., Emmerling M., Fiebich B.L. (2000). Inflammation and Alzheimer’s disease. Neurobiol. Aging.

[107] Farlow M.R., Miller M.L., Pejovic V. (2008). Treatment Options in Alzheimer’s Disease: Maximizing Benefit, Managing Expectations. Dement. Geriatr. Cogn. Disord..

[108] Jiao Y.B., Rui Y.C., Yang P.Y., Li T.J., Qiu Y. (2007). Effects of *Ginkgo biloba* extract on expressions of IL-1beta, TNF-alpha, and IL-10 in U937 foam cells. Yao Xue Xue Bao.

[109] Samandar F., Tehranizadeh Z.A., Saberi M.R., Chamani J. (2022). CB1 as a novel target for *Ginkgo biloba*’s terpene trilactone for controlling chemotherapy-induced peripheral neuropathy (CIPN). J. Mol. Model..

[110] Bampidis V., Azimonti G., Bastos M.L., Christensen H., Durjava M., Kouba M., López-Alonso M., López Puente S., Marcon F., Mayo B. (2024). Safety and efficacy of feed additives consisting of ginkgo tinctures obtained from the leaves of *Ginkgo biloba* L. for use in all animal species (FEFANA asbl). EFSA J. Eur. Food Saf. Auth..

[111] Koh P.O. (2011). Identification of proteins differentially expressed in cerebral cortexes of *Ginkgo biloba* extract (EGb761)-treated rats in a middle cerebral artery occlusion model--a proteomics approach. Am. J. Chin. Med..

[112] Meng M., Ai D., Sun L., Xu X., Cao X. (2019). EGb 761 inhibits Aβ1-42-induced neuroinflammatory response by suppressing P38 MAPK signaling pathway in BV-2 microglial cells. Neuroreport.

[113] Yadav N., Palkhede J.D., Kim S.Y. (2023). Anti-Glucotoxicity Effect of Phytoconstituents via Inhibiting MGO-AGEs Formation and Breaking MGO-AGEs. Int. J. Mol. Sci..

[114] Huang C.Y., Deng J.S., Huang W.C., Jiang W.P., Huang G.J. (2020). Attenuation of Lipopolysaccharide-Induced Acute Lung Injury by Hispolon in Mice, Through Regulating the TLR4/PI3K/Akt/mTOR and Keap1/Nrf2/HO-1 Pathways, and Suppressing Oxidative Stress-Mediated ER Stress-Induced Apoptosis and Autophagy. Nutrients.

[115] Zhang L., Li G., Tao S., Xia P., Chaudhry N., Kaura S., Stone S.S., Liu M. (2022). Ginkgo Biloba Extract Reduces Cardiac and Brain Inflammation in Rats Fed a HFD and Exposed to Chronic Mental Stress through NF-κB Inhibition. Mediat. Inflamm..

[116] Tonnesen M.G., Anderson D.C., Springer T.A., Knedler A., Avdi N., Henson P.M. (1989). Adherence of neutrophils to cultured human microvascular endothelial cells. Stimulation by chemotactic peptides and lipid mediators and dependence upon the Mac-1, LFA-1, p150,95 glycoprotein family. J. Clin. Investig..

[117] Wang L.T., Huang H., Chang Y.H., Wang Y.Q., Wang J.D., Cai Z.H., Efferth T., Fu Y.J. (2022). Biflavonoids from *Ginkgo biloba* leaves as a novel anti-atherosclerotic candidate: Inhibition potency and mechanistic analysis. Phytomedicine.

[118] Jiao Y.B., Rui Y.C., Li T.J., Yang P.Y., Qiu Y. (2005). Expression of pro-inflammatory and anti-inflammatory cytokines in brain of atherosclerotic rats and effects of *Ginkgo biloba* extract. Acta Pharmacol. Sin..

[119] Fu Z., Lin L., Liu S., Qin M., He S., Zhu L., Huang J. (2019). Ginkgo Biloba Extract Inhibits Metastasis and ERK/Nuclear Factor kappa B (NF-κB) Signaling Pathway in Gastric Cancer. Med. Sci. Monit..

[120] Koh P.O. (2009). *Gingko biloba* extract (EGb 761) prevents increase of Bad-Bcl-XL interaction following cerebral ischemia. Am. J. Chin. Med..

[121] Duhamel T.A., Xu Y.-J., Arneja A.S., Dhalla N.S. (2007). Targeting platelets for prevention and treatment of cardiovascular disease. Expert. Opin. Ther. Targets.

[122] Chudhary M., Zhang C., Song S., Ren X., Kong L. (2021). *Ginkgo biloba* delays light-induced photoreceptor degeneration through antioxidant and antiapoptotic properties. Exp. Ther. Med..

[123] Li Z., Xiao G., Wang H., He S., Zhu Y. (2021). A preparation of *Ginkgo biloba* L. leaves extract inhibits the apoptosis of hippocampal neurons in post-stroke mice via regulating the expression of Bax/Bcl-2 and Caspase-3. J. Ethnopharmacol..

[124] Teleanu D.M., Niculescu A.G., Lungu I.I., Radu C.I., Vladâcenco O., Roza E., Costăchescu B., Grumezescu A.M., Teleanu R.I. (2022). An Overview of Oxidative Stress, Neuroinflammation, and Neurodegenerative Diseases. Int. J. Mol. Sci..

[125] Lushchak V.I. (2014). Free radicals, reactive oxygen species, oxidative stress and its classification. Chem. Biol. Interact..

[126] Forman H.J., Zhang H. (2021). Targeting oxidative stress in disease: Promise and limitations of antioxidant therapy. Nat. Rev. Drug Discov..

[127] Dickinson B.C., Chang C.J. (2011). Chemistry and biology of reactive oxygen species in signaling or stress responses. Nat. Chem. Biol..

[128] Chatgilialoglu C., Ferreri C., Krokidis M.G., Masi A., Terzidis M.A. (2021). On the relevance of hydroxyl radical to purine DNA damage. Free Radic. Res..

[129] Smirnoff N., Arnaud D. (2019). Hydrogen peroxide metabolism and functions in plants. New Phytol..

[130] Wong H.S., Dighe P.A., Mezera V., Monternier P.A., Brand M.D. (2017). Production of superoxide and hydrogen peroxide from specific mitochondrial sites under different bioenergetic conditions. J. Biol. Chem..

[131] Neha K., Haider M.R., Pathak A., Yar M.S. (2019). Medicinal prospects of antioxidants: A review. Eur. J. Med. Chem..

[132] Lu Y., Rong J., Lai Y., Tao L., Yuan X., Shu X. (2020). The Degree of Helicobacter pylori Infection Affects the State of Macrophage Polarization through Crosstalk between ROS and HIF-1alpha. Oxid. Med. Cell Longev..

[133] Tamura H., Jozaki M., Tanabe M., Shirafuta Y., Mihara Y., Shinagawa M., Tamura I., Maekawa R., Sato S., Taketani T. (2020). Importance of Melatonin in Assisted Reproductive Technology and Ovarian Aging. Int. J. Mol. Sci..

[134] Mezhnina V., Ebeigbe O.P., Poe A., Kondratov R.V. (2022). Circadian Control of Mitochondria in Reactive Oxygen Species Homeostasis. Antioxid. Redox Signal..

[135] Feduska J.M., Tse H.M. (2018). The proinflammatory effects of macrophage-derived NADPH oxidase function in autoimmune diabetes. Free Radic. Biol. Med..

[136] Brieger K., Schiavone S., Miller F.J., Krause K.H. (2012). Reactive oxygen species: From health to disease. Swiss Med. Wkly..

[137] Milisav I., Ribarič S., Poljsak B. (2018). Antioxidant Vitamins and Ageing. Subcell Biochem..

[138] Hass D.T., Barnstable C.J. (2021). Uncoupling proteins in the mitochondrial defense against oxidative stress. Prog. Retin. Eye Res..

[139] Glorieux C., Calderon P.B. (2017). Catalase, a remarkable enzyme: Targeting the oldest antioxidant enzyme to find a new cancer treatment approach. Biol. Chem..

[140] Azzu V., Jastroch M., Divakaruni A.S., Brand M.D. (2010). The regulation and turnover of mitochondrial uncoupling proteins. Biochim. Biophys. Acta.

[141] Zhu L., Li Z., Sheng L., Zhang F., Ji W. (2024). Ginkgolide A attenuated apoptosis via inhibition of oxidative stress in mice with traumatic brain injury. Heliyon.

[142] Nguyen T., Alzahrani T. (2024). Ginkgo Biloba. StatPearls.

[143] Mohammadi Zonouz A., Ghasemzadeh Rahbardar M., Hosseinzadeh H. (2024). The molecular mechanisms of ginkgo (*Ginkgo biloba*) activity in signaling pathways: A comprehensive review. Phytomedicine.

[144] Pu X., Fu Y., Yang Y., Xu G. (2024). *Ginkgo biloba* extract alleviates CCl(4)-induced acute liver injury by regulating PI3K/AKT signaling pathway. Heliyon.

[145] Huang Z., Yuan T., Chen J., Jiang M., Yan R., Yang W., Wang L., Liao Y., Huang G. (2022). Neuroprotective and antioxidant activities of different polarity parts of the extracts of the *Ginkgo biloba* leaf and *Zingiber officinale* rhizome from Yongzhou. Front. Chem..

[146] Liu Q., Jin Z., Xu Z., Yang H., Li L., Li G., Li F., Gu S., Zong S., Zhou J. (2019). Antioxidant effects of ginkgolides and bilobalide against cerebral ischemia injury by activating the Akt/Nrf2 pathway in vitro and in vivo. Cell Stress Chaperones.

[147] Klomsakul P., Aiumsubtub A., Chalopagorn P. (2022). Evaluation of Antioxidant Activities and Tyrosinase Inhibitory Effects of *Ginkgo biloba* Tea Extract. ScientificWorldJournal.

[148] Achete de Souza G., de Marqui S.V., Matias J.N., Guiguer E.L., Barbalho S.M. (2020). Effects of *Ginkgo biloba* on Diseases Related to Oxidative Stress. Planta Med..

[149] Sarkar C., Quispe C., Jamaddar S., Hossain R., Ray P., Mondal M., Abdulwanis Mohamed Z., Sani Jaafaru M., Salehi B., Islam M.T. (2020). Therapeutic promises of ginkgolide A: A literature-based review. Biomed. Pharmacother..

[150] Strømgaard K., Nakanishi K. (2004). Chemistry and biology of terpene trilactones from *Ginkgo biloba*. Angew. Chem. (Int. Ed. Engl.).

[151] Tao Z., Jin W., Ao M., Zhai S., Xu H., Yu L.J.F. (2019). Evaluation of the anti-inflammatory properties of the active constituents in *Ginkgo biloba* for the treatment of pulmonary diseases. Food Funct..

[152] Tian J., Liu Y., Liu Y., Chen K., Lyu S.J.O. (2018). *Ginkgo biloba* leaf extract protects against myocardial injury via attenuation of endoplasmic reticulum stress in streptozotocin-induced diabetic ApoE^−/−^ mice. Oxidative Med. Cell. Longev..

[153] Trebaticka J., Ďuračková Z. (2015). Psychiatric disorders and polyphenols: Can they be helpful in therapy?. Oxidative Med. Cell. Longev..

[154] Choudhary S., Kumar P., Malik J.J.P.R. (2013). Plants and phytochemicals for Huntington’s disease. Pharmacogn. Rev..

[155] Unger M. (2013). Pharmacokinetic drug interactions involving *Ginkgo biloba*. Drug Metab. Rev..

[156] Saini A.S., Taliyan R., Sharma P.L.J.P. (2014). Protective effect and mechanism of *Ginkgo biloba* extract-EGb 761 on STZ-induced diabetic cardiomyopathy in rats. Pharmacogn. Mag..

[157] Wang L., Bai Y., Wang B., Cui H., Wu H., Lv J.-R., Mei Y., Zhang J.-S., Liu S., Qi L.-W.J. (2013). Suppression of experimental abdominal aortic aneurysms in the mice by treatment with *Ginkgo biloba* extract (EGb 761). J. Ethnopharmacol..

[158] Wang A., Yang Q., Li Q., Wang X., Hao S., Wang J., Ren M. (2018). Ginkgo Biloba L. extract reduces H_2_O_2_-induced bone marrow mesenchymal stem cells cytotoxicity by regulating mitogen-activated protein kinase (MAPK) signaling pathways and oxidative stress. Med. Sci. Monit. Int. Med. J. Exp. Clin. Res..

[159] Wang C., Wang B.J.P.R. (2016). *Ginkgo biloba* extract attenuates oxidative stress and apoptosis in mouse cochlear neural stem cells. Phytotherapy Res..

[160] Kaur S., Sharma N., Nehru B.J.I. (2018). Anti-inflammatory effects of *Ginkgo biloba* extract against trimethyltin-induced hippocampal neuronal injury. Inflammopharmacology.

[161] Mueller J.K., Muller W.E. (2024). Multi-target drugs for the treatment of cognitive impairment and fatigue in post-COVID syndrome: Focus on *Ginkgo biloba* and *Rhodiola rosea*. J. Neural. Transm. (Vienna).

[162] Zou H., Fang J., Han Y., Hu X., Meng J., Huang F., Xu H., Lu C., Wang Y., Zhang L. (2023). Effects and safety of *Ginkgo biloba* on blood metabolism in type 2 diabetes mellitus: A systematic review and meta-analysis. Front. Endocrinol..

[163] Ni Q., Zhu T., Wang W., Guo D., Li Y., Chen T., Zhang X. (2024). Green Synthesis of Narrow-Size Silver Nanoparticles Using *Ginkgo biloba* Leaves: Condition Optimization, Characterization, and Antibacterial and Cytotoxic Activities. Int. J. Mol. Sci..

[164] Yang T., Du X., Xu L. (2024). Radioprotective effect of Ginkgolide B on brain: The mediating role of DCC/MST1 signaling. Int. J. Radiat. Biol..

[165] Li Y., Wu Y., Yao X., Hao F., Yu C., Bao Y., Wu Y., Song Z., Sun Y., Zheng L.J. (2017). Ginkgolide A ameliorates LPS-induced inflammatory responses in vitro and in vivo. Int. J. Mol. Sci..

[166] Zhang W., Song J.K., Yan R., Li L., Xiao Z.Y., Zhou W.X., Wang Z.Z., Xiao W., Du G.H. (2018). Diterpene ginkgolides protect against cerebral ischemia/reperfusion damage in rats by activating Nrf2 and CREB through PI3K/Akt signaling. Acta Pharmacol. Sin..

[167] Chen W.S.-T., Lin T.-Y., Kuo C.-H., Hsieh D.J.-Y., Kuo W.-W., Liao S.-C., Kao H.-C., Ju D.-T., Lin Y.-J., Huang C.-Y.J.A. (2023). Ginkgolide A improves the pleiotropic function and reinforces the neuroprotective effects by mesenchymal stem cell-derived exosomes in 6-OHDA-induced cell model of Parkinson’s disease. Aging.

[168] Wang J., Zhuang L., Ding Y., Wang Z., Xiao W., Zhu J. (2021). A RNA-seq approach for exploring the protective effect of ginkgolide B on glutamate-induced astrocytes injury. J. Ethnopharmacol..

[169] Zheng P.-D., Mungur R., Zhou H.-J., Hassan M., Jiang S.-N., Zheng J. (2018). Ginkgolide B promotes the proliferation and differentiation of neural stem cells following cerebral ischemia/reperfusion injury, both in vivo and in vitro. Neural Regen. Res..

[170] Wang L., Lei Q., Zhao S., Xu W., Dong W., Ran J., Shi Q., Fu J.J. (2021). Ginkgolide B maintains calcium homeostasis in hypoxic hippocampal neurons by inhibiting calcium influx and intracellular calcium release. Front. Cell. Neurosci..

[171] Liou C.-J., Lai X.-Y., Chen Y.-L., Wang C.-L., Wei C.-H., Huang W.-C.J. (2015). Ginkgolide C suppresses adipogenesis in 3T3-L1 adipocytes via the AMPK signaling pathway. Evid.-Based Complement. Altern. Med..

[172] Tchantchou F., Lacor P.N., Cao Z., Lao L., Hou Y., Cui C., Klein W.L., Luo Y.J. (2009). Stimulation of neurogenesis and synaptogenesis by bilobalide and quercetin via common final pathway in hippocampal neurons. J. Alzheimer’s Dis..

[173] Shi C., Wu F., Xu J., Zou J.J. (2011). Bilobalide regulates soluble amyloid precursor protein release via phosphatidyl inositol 3 kinase-dependent pathway. Neurochem. Int..

[174] Wang H., Jiang T., Li W., Gao N., Zhang T. (2018). Resveratrol attenuates oxidative damage through activating mitophagy in an in vitro model of Alzheimer’s disease. Toxicol. Lett..

[175] Zhao H., Li N., Wang Q., Cheng X., Li X., Liu T.J.N. (2015). Resveratrol decreases the insoluble Aβ1–42 level in hippocampus and protects the integrity of the blood–brain barrier in AD rats. Neuroscience.

[176] Foudah A.I., Devi S., Alam A., Salkini M.A., Ross S.A. (2023). Anticholinergic effect of resveratrol with vitamin E on scopolamine-induced Alzheimer’s disease in rats: Mechanistic approach to prevent inflammation. Front. Pharmacol..

[177] Tanaka M., Vécsei L.J.B. (2015). A Decade of Dedication: Pioneering Perspectives on Neurological Diseases and Mental Illnesses. Multidisciplinary Digital Publishing Institute: 2024; Vol. 12, p 1083.Fang, X.; Jiang, Y.; Ji, H.; Zhao, L.; Xiao, W.; Wang, Z.; Ding, G. The synergistic beneficial effects of Ginkgo flavonoid and Coriolus versicolor polysaccharide for memory improvements in a mouse model of dementia. Evid. Based Complement Alternat Med..

[178] Chopin P., Briley M. (1992). Effects of four non-cholinergic cognitive enhancers in comparison with tacrine and galanthamine on scopolamine-induced amnesia in rats. Psychopharmacology.

[179] Weingartner H. (1985). Models of memory dysfunctions. Ann. N. Y. Acad. Sci..

[180] Kristofiková Z., Klaschka J. (1997). In vitro effect of *Ginkgo biloba* extract (EGb 761) on the activity of presynaptic cholinergic nerve terminals in rat hippocampus. Dement. Geriatr. Cogn. Disord..

[181] Winter E. (1991). Effects of an extract of *Ginkgo biloba* on learning and memory in mice. Pharmacol. Biochem. Behav..

[182] Gajewski A., Hensch S.A. (1999). *Ginkgo biloba* and memory for a maze. Psychol. Rep..

[183] Rigney U., Kimber S., Hindmarch I. (1999). The effects of acute doses of standardized *Ginkgo biloba* extract on memory and psychomotor performance in volunteers. Phytother. Res. PTR.

[184] Rai G.S., Shovlin C., Wesnes K.A. (1991). A double-blind, placebo controlled study of *Ginkgo biloba* extract (t‘anakan’) in elderly outpatients with mild to moderate memory impairment. Curr. Med. Res. Opin..

[185] Fan F., Liu H., Shi X., Ai Y., Liu Q., Cheng Y. (2022). The Efficacy and Safety of Alzheimer’s Disease Therapies: An Updated Umbrella Review. J. Alzheimer’s Dis. JAD.

[186] Willard S.S., Koochekpour S. (2013). Glutamate, glutamate receptors, and downstream signaling pathways. Int. J. Biol. Sci..

[187] Lundstrom K., Pham H.T., Dinh L.D. (2017). Interaction of Plant Extracts with Central Nervous System Receptors. Medicines.

[188] Yoshitake T., Yoshitake S., Kehr J. (2010). The *Ginkgo biloba* extract EGb 761^®^ and its main constituent flavonoids and ginkgolides increase extracellular dopamine levels in the rat prefrontal cortex. Br. J. Pharmacol..

[189] Tavares R.G. (2005). Modulação do Sistema Glutamatérgico: Estudo dos Efeitos do ácido Quinolínico e dos Derivados da Guanina. http://hdl.handle.net/10183/12714.

[190] Hornykiewicz O. (1966). Dopamine (3-hydroxytyramine) and brain function. Pharmacol. Rev..

[191] Yeh K.-Y., Wu C.-H., Tai M.-Y., Tsai Y.-F. (2011). *Ginkgo biloba* extract enhances noncontact erection in rats: The role of dopamine in the paraventricular nucleus and the mesolimbic system. Neuroscience.

[192] Tian J., Guo L., Sui S., Driskill C., Phensy A., Wang Q., Gauba E., Zigman J.M., Swerdlow R.H., Kroener S. (2019). Disrupted hippocampal growth hormone secretagogue receptor 1α interaction with dopamine receptor D1 plays a role in Alzheimer′ s disease. Sci. Transl. Med..

[193] Butters M.A., Young J.B., Lopez O., Aizenstein H.J., Mulsant B.H., Reynolds C.F., DeKosky S.T., Becker J.T. (2008). Pathways linking late-life depression to persistent cognitive impairment and dementia. Dialogues Clin. Neurosci..

[194] Koyama T., Nakajima Y., Fujii T., Kawashima K. (1999). Enhancement of cortical and hippocampal cholinergic neurotransmission through 5-HT1A receptor-mediated pathways by BAY x 3702 in freely moving rats. Neurosci. Lett..

[195] Baranger K., Giannoni P., Girard S.D., Girot S., Gaven F., Stephan D., Migliorati M., Khrestchatisky M., Bockaert J., Marchetti-Gauthier E. (2017). Chronic treatments with a 5-HT(4) receptor agonist decrease amyloid pathology in the entorhinal cortex and learning and memory deficits in the 5xFAD mouse model of Alzheimer’s disease. Neuropharmacology.

[196] Kehr J., Yoshitake S., Ijiri S., Koch E., Nöldner M., Yoshitake T. (2012). *Ginkgo biloba* leaf extract (EGb 761^®^) and its specific acylated flavonol constituents increase dopamine and acetylcholine levels in the rat medial prefrontal cortex: Possible implications for the cognitive enhancing properties of EGb 761^®^. Int. Psychogeriatr..

[197] Dillon K.A., Gross-Isseroff R., Israeli M., Biegon A. (1991). Autoradiographic analysis of serotonin 5-HT1A receptor binding in the human brain postmortem: Effects of age and alcohol. Brain Res..

[198] Huguet F., Drieu K., Piriou A. (1994). Decreased cerebral 5-HT1A receptors during ageing: Reversal by *Ginkgo biloba* extract (EGb 761). J. Pharm. Pharmacol..

[199] Kandiah N., Chan Y.F., Chen C., Dasig D., Dominguez J., Han S.H., Jia J., Kim S., Limpawattana P., Ng L.L. (2021). Strategies for the use of *Ginkgo biloba* extract, EGb 761®, in the treatment and management of mild cognitive impairment in Asia: Expert consensus. CNS Neurosci. Ther..

[200] Shi C., Liu J., Wu F., Yew D.T. (2010). *Ginkgo biloba* extract in Alzheimer’s disease: From action mechanisms to medical practice. Int. J. Mol. Sci..

[201] Shahidi S., Asl S.S., Komaki A., Hashemi-Firouzi N. (2018). The effect of chronic stimulation of serotonin receptor type 7 on recognition, passive avoidance memory, hippocampal long-term potentiation, and neuronal apoptosis in the amyloid β protein treated rat. Psychopharmacology.

[202] Rojas P., Rojas C., Ebadi M., Montes S., Monroy-Noyola A., Serrano-García N. (2004). EGb761 pretreatment reduces monoamine oxidase activity in mouse corpus striatum during 1-methyl-4-phenylpyridinium neurotoxicity. Neurochem. Res..

[203] Blecharz-Klin K., Piechal A., Joniec I., Pyrzanowska J., Widy-Tyszkiewicz E. (2009). Pharmacological and biochemical effects of *Ginkgo biloba* extract on learning, memory consolidation and motor activity in old rats. Acta Neurobiol. Exp..

[204] Schneider L.S., DeKosky S.T., Farlow M.R., Tariot P.N., Hoerr R., Kieser M. (2005). A randomized, double-blind, placebo-controlled trial of two doses of *Ginkgo biloba* extract in dementia of the Alzheimer’s type. Curr. Alzheimer Res..

[205] Porciúncula L.O., Goto-Silva L., Ledur P.F., Rehen S.K. (2021). The Age of Brain Organoids: Tailoring Cell Identity and Functionality for Normal Brain Development and Disease Modeling. Front. Neurosci..

[206] Giorgi C., Lombardozzi G., Ammannito F., Scenna M.S., Maceroni E., Quintiliani M., d‘Angelo M., Cimini A., Castelli V. (2024). Brain Organoids: A Game-Changer for Drug Testing. Pharmaceutics.

[207] Daly B.P., Eichen D.M., Bailer B., Brown R.T., Buchanan C.L., Ramachandran V.S. (2012). Central Nervous System. Encyclopedia of Human Behavior.

[208] Lavazza A. (2021). ‘Consciousnessoids’: Clues and insights from human cerebral organoids for the study of consciousness. Neurosci. Conscious.

[209] Qian X., Jacob F., Song M.M., Nguyen H.N., Song H., Ming G.L. (2018). Generation of human brain region-specific organoids using a miniaturized spinning bioreactor. Nat. Protoc..

[210] Bathina S., Das U.N. (2015). Brain-derived neurotrophic factor and its clinical implications. Arch. Med. Sci..

[211] Mansour A.A., Gonçalves J.T., Bloyd C.W., Li H., Fernandes S., Quang D., Johnston S., Parylak S.L., Jin X., Gage F.H. (2018). An in vivo model of functional and vascularized human brain organoids. Nat. Biotechnol..

[212] Cacciamali A., Villa R., Dotti S. (2022). 3D Cell Cultures: Evolution of an Ancient Tool for New Applications. Front. Physiol..

[213] Sreenivasamurthy S., Laul M., Zhao N., Kim T., Zhu D. (2023). Current progress of cerebral organoids for modeling Alzheimer’s disease origins and mechanisms. Bioeng. Transl. Med..

[214] Amirifar L., Shamloo A., Nasiri R., de Barros N.R., Wang Z.Z., Unluturk B.D., Libanori A., Ievglevskyi O., Diltemiz S.E., Sances S. (2022). Brain-on-a-chip: Recent advances in design and techniques for microfluidic models of the brain in health and disease. Biomaterials.

[215] Park J., Lee B.K., Jeong G.S., Hyun J.K., Lee C.J., Lee S.H. (2015). Three-dimensional brain-on-a-chip with an interstitial level of flow and its application as an in vitro model of Alzheimer’s disease. Lab. Chip.

